# Time-Delayed Mutual Information of the Phase as a Measure of Functional Connectivity

**DOI:** 10.1371/journal.pone.0044633

**Published:** 2012-09-18

**Authors:** Andreas Wilmer, Marc de Lussanet, Markus Lappe

**Affiliations:** 1 Department of Psychology, Otto-Creutzfeldt Center for Cognitive and Behavioral Neuroscience (OCC), Westfälische Wilhelms-Universität, Münster, Germany; Wake Forest School of Medicine, United States of America

## Abstract

We propose a time-delayed mutual information of the phase for detecting nonlinear synchronization in electrophysiological data such as MEG. Palus already introduced the mutual information as a measure of synchronization [1]. To obtain estimates on small data-sets as reliably as possible, we adopt the numerical implementation as proposed by Kraskov and colleagues [2]. An embedding with a parametric time-delay allows a reconstruction of arbitrary nonstationary connective structures – so-called connectivity patterns – in a wide class of systems such as coupled oscillatory or even purely stochastic driven processes [3]. By using this method we do not need to make any assumptions about coupling directions, delay times, temporal dynamics, nonlinearities or underlying mechanisms. For verifying and refining the methods we generate synthetic data-sets by a mutual amplitude coupled network of Rössler oscillators with an a-priori known connective structure. This network is modified in such a way, that the power-spectrum forms a 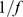 power law, which is also observed in electrophysiological recordings. The functional connectivity measure is tested on robustness to additive uncorrelated noise and in discrimination of linear mixed input data. For the latter issue a suitable de-correlation technique is applied. Furthermore, the compatibility to inverse methods for a source reconstruction in MEG such as beamforming techniques is controlled by dedicated dipole simulations. Finally, the method is applied on an experimental MEG recording.

## Introduction

In cognitive neuroscience a central goal is the understanding of processing information in the brain. Two basic questions of general interest can be addressed: First, what are the underlying mechanisms of cortical communication and second, how is information of the environment processed? It has become clear that synchronization plays a fundamental role in cortical processing as a partial answer to fundamental cortical mechanisms of communication [4]–[9]. To fill the gap between low-level neuronal mechanisms and cognition, a wide diversity of methods have been developed for recording and imaging the active brain. The methods cover a huge range of scales ranging from single-unit recordings on a microscopic level to whole brain imaging techniques such as fMRI, EEG and MEG [10], [11]. Each technique has its advantages and disadvantages and offers just a partial view of the complete system. Recording techniques with high temporal resolution such as EEG and MEG are suitable for measuring oscillatory dynamics in the human brain. Both methods feature a non-invasive data acquisition and measure the primary electrical neuronal response of a population with high temporal accuracy (albeit low spatial precision and accuracy). In the present study, we are especially interested in the interaction between different cortical areas in terms of their oscillatory dynamics and particularly in the synchronization among brain areas. The phenomenon of synchronization is investigated in the field of nonlinear and chaotic systems [12]–[14]. A huge battery of tools for the quantification of synchronization in the field of neuroscience on diverse temporal and spatial scales is available. Le van Quyen, Bragin [15] and Sakkalis [16] summarize several common data-driven concepts. Analogously to the cross-correlation the *coherency* is sensitive to linear interactions among two signals in the frequency domain [17]. A class of techniques known as as *nested oscillations*, detects couplings among amplitudes and phases among frequencies belonging to cross-frequency couplings (CFC) [18] or the *phase coherence* (or phase locked value, PLV) [19]–[22]. The *synchronization index* [23] constitute a measure of phase synchronization. These measures are sensitive to a specified order of synchronization n∶m (CFC type). However, it is not fully understood how a cortical network and the underlying dynamics are related, in particular which temporal and spatial scales are sufficient to describe the system [24]–[26] and how a mental state is linked to a corresponding activation pattern in terms of a transient dynamics [27].

Our main goal in this work is to embed the mutual information of the phase in our framework of a functional connectivity analysis in terms of phase synchronization [3]. We are striving for a universal analysis tool capable of assessing the synchronization of a system in which the fundamental dynamics are not well-known, i.e. such requirements effort a technique respecting on the one hand a nonstationarity of the underlying system and on the other hand being sensitive to both linear and nonlinear interactions [24]–[26]. Many studies have estimated synchrony between sensor pairs e.g. [5], [23], [28], [29]. In our study we want to focus on the analysis of reconstructed cortical sources e.g. [30], [31]. To make our approach applicable to such data we implement and validate the following aspects:

1. We customize the mutual information for trial based MEG data-sets. This allows a time-dependent analysis of phase synchronization of bivariate time-series with an underlying nonstationary dynamics from trial based data. We do not enter any information about the direction of coupling, the temporal dynamics, the amount of time-delays nor the order of synchronization. As a result a connectivity pattern is assembled for each bivariate data-set, which contains the coupling strength of two sources as a function of time and time-delay in terms of nonlinear correlations.
2. We suggest a simple but efficient method for suppressing artifacts generated by partially correlated time-series, which form a well-known issue in reconstructed cortical sources. The artifact reduction is tested by using a statistical validation and synthetic data with a prior known coupling structure.
3. We check our approach for compatibility with beamforming source reconstructions by the simulation of cortical sources in MEG.
4. The method is tested with an experimental MEG paradigm by Steinberg and colleagues on processing of emotionally relevant stimuli [32].

In the Methods time-delayed mutual information of the phase is explained [1]. Analogously to the phase measures of synchronization in [3] this measure is designed to be applied to trial based bivariate data-sets. To assure an estimation with high data efficiency and accuracy we adopt the implementation of [2]. Next, we generate synthetic data-sets with a-priori known connective structures for testing purposes. We assemble a simple network with time and delay dependent linear couplings based on Rössler oscillators as already presented previously in [3]. The Rössler oscillator is a nonlinear system holding an oscillatory chaotic dynamics. It is used as a standard model for the investigation of synchronization [13] and is also very common for testing new techniques of data analysis in the field of neuroscience [31], [33]–[36]. Further, we modify the Rössler network resulting in a 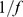 power law in the power-spectrum, which can be phenomenologically observed in EEG recordings [25], [37], [38]. Synthetic data from both the unmodified oscillatory and the broadband 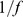 system is used for testing and refining our methods.

The Results are divided into three parts: In the first part we benchmark the mutual information. Therefore, we address the issue of partially mixed time-series by varying a linear superposition of two data-sets. Correlated data is typically observed in imperfect reconstructions of cortical sources. Additionally, we explore the robustness of the analysis outcome on data contaminated with additive noise, which is usually a result of thermal effects in MEG recordings [39]. We suggest a de-correlation procedure of the data on image level by correcting the connectivity pattern directly. Next, we compare our de-correlation procedure with a de-mixing on data level with the help of an independent component analysis (ICA) [40] of the correlated time-series. We also investigate the effect of the number of trials on the analysis outcome and compare it qualitatively to the amplitude cross-correlation, the phase coherence and the phase synchronization. A statistical rating is achieved by a false discovery control (FDR) following the suggestion of [41], [42].

The second part of the results focuses on simulated MEG data-sets by using the Fieldtrip toolbox for matlab [43]. Hadjipapas et al. proved the principle compatibility of a source reconstruction by beamforming with phase sensitive techniques [31]. However, they used estimations across time and trials. Our purpose is to enter explicitly the underlying nonstationary dynamics. Thus, we aim to refine our approach based on a time-dependent estimate of the mutual information by combining it with our proposed de-correlation procedure. In the first step an MEG recording is simulated after placing cortical sources with a prior known coupling. In a second step the sources are reconstructed subsequently from the simulated data-set using a beamformer. We want to clarify if the basic assumptions of the beamformer technique – a statistical independency of non-delayed neuronal activities [44] – conflict with our method of investigating synchronicity for two reasons: first, a suppression of linear correlated sources might be problematic regarding the detection of phase synchrony at first sight and second, the mapping of the data might distort or destroy the modeled correlations of the phases. In a pre-processing step the beamforming results are improved by a noise reduction using a linear weighted moving average (WMA), which is applied on the simulated data in the sensor space before the beamforming to improve the results of the reconstructed connectivity. We simulate and analyze the synthetic MEG data-sets with varying the number of trials and the amount of thermal sensor noise and find a much better stability of the reproduced connectivity patterns when a noise reduction is applied before.

In the last part we apply the functional connectivity analysis to an MEG group study by Steinberg and colleagues on processing of emotionally relevant stimuli [32]. In their paradigm subjects were conditioned with hydrogen sulfide while watching faces with a neutral expression. Steinberg and colleagues found an early change in activity at 50–80 ms in frontal and temporal regions. We estimate the synchronicity between both regions across the subjects and are able to recover a significant correlation between them providing evidence that our method can be applied to real MEG recordings.

## Methods

### Estimation of the phase

The concept of phase is well-known in literature [13], [14]. There are different ways to estimate the phase of time-series. [45] compared a complex wavelet transform with a Hilbert transform for the analysis of neuronal data and found no essential differences. Because we are interested in the mutual information of the phase, the phase has to be estimated from the amplitude time-series. The instantaneous phase 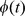 of a signal 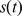 can be estimated using the Hilbert transform 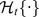:
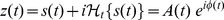
The analytic signal 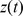 can be understood as an embedding of the one dimensional time-series in the two dimensional complex plane. The cyclic phase is computed by the following expression:
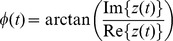
In the following the cyclic variable 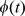 is defined in such a way that it is periodic in the interval 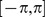. Strictly speaking the phase of a signal is only defined in a physically meaningful manner if the spectrum of a signal is narrow-banded [45], [46]. In [3] we already showed that synchronous states can even be discovered successfully in white noise driven, amplitude coupled Ornstein-Uhlenbeck processes, which are broad-banded and purely stochastic processes.

### Mutual information of the phase

Rosenblum and colleagues found in their work that the phenomenon of synchronization can be described as a certain phase relation between interacting systems [13]. There are many ways to measure synchronization between processes, see e.g. [15], [46]. In neuroscience it is usually assumed that synchronized systems are connected. One can discriminate between effective and functional connectivity [47]. A functional connectivity typically denotes a statistical property in terms of a correlative relation between brain areas, whereas an effective connectivity describes a directed influence among neuronal assemblies in terms of a driver response relationship.

In our approach we want to address the mutual information of the phase [1]. The mutual information is a model-free measure of the shared information among two stochastic variables in terms of a nonlinear correlation, i.e. also correlations of second or higher order [48]. Its correlative nature makes it belong to the class of functional connectivities. Thus, it generally forms a symmetric measure of correlation, which is invariant to the commutation of the input, i.e. the direction of the covariation is not distinguishable between the two variables. To break the symmetry we expand the mutual information by a parametric time-delay, which makes it sensitive to the direction of the covariation given by the temporal order.

There are several reasons why we implement the mutual information of the phase as a measure of synchronization: First, we follow a data-driven approach because we want to make as few prior assumptions as possible. With the mutual information all orders n:m as well as nonlinear relations are included and quantified simultaneously. Second, we decided to focus on the signal phases and not amplitudes because in the investigation of synchronization, relations between phases are more natural than relations between amplitudes [14]. In cross-correlations of the amplitude one has to deal with oscillations in the correlation function, if the spectrum of the data is narrow-banded. If phases are used instead of amplitudes the outcome of the correlation is smooth in both narrow- and broad-banded signals [3]. Third, because the mutual information covers the correlations across all frequencies implicitly, the solution space is reduced by two dimensions compared to CFC based techniques. Fourth, we want to enter the time-dependent transient interactions between signals, i.e. nonstationary processes can be analyzed. This requires an estimation of the synchronization measure from data-sets across trials. As we will see later the estimation of the mutual information from trial-based data-sets of the size of a typical MEG recording is a challenging but still feasible task. Finally, as we follow a data-driven approach the estimated results are independent on prior assumptions such as an initial configuration of a network or the type of interactions within such networks. Two elements within a certain network are analyzed independently and pairwise, i.e. an expansion of the network by including new sources does not influence the previous results. Because of the conceptual independency of the elements there is no risk of an overfitting.

In our approach the flow of information between two distinct regions indexed with 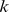 and 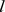 is addressed by estimating the phase synchronization of bivariate time-series. In the following the terms of driver and response denote a delayed covariation among two variables, whereas the driver is defined with the index 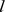 and the responder with 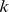. The driving system 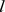 is shifted back in time with 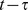 and a time lag of 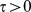 compared to the non-delayed driven system 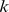. The mutual information 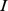 forms a non-negative dependency measure, which equals zero in the case of independency. Applied on phases it forms a measure of synchronicity [1]. Regarding the time dependency and a time-delay 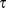 it is given for the phases 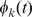 and 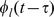 by the expression:
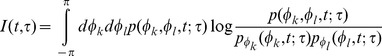
(1)with the marginal densities 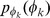, 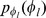 and the formal expression of the joint probability density:
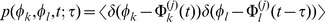
(2)Thereby 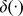 denotes the Dirac delta function and 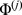 a sampled data-point corresponding to a specific time of the 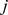
th trial. Further, we use the abbreviation 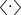 for a combined trial averaging and moving time window with a window size of 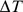, i.e. the sample points across the trials and within the time interval 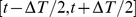 are pooled together. The joint entropy 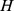 gives the total common information of both signals, which marks the upper bound for the shared information. As in Eq. (1) we consider it as an explicit function of the time and the time-delay:
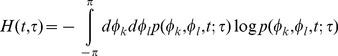
(3)We can use the joint entropy 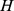 for a normalization of 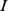 dividing Eq. (1) simply by Eq. (3). This leads to a bounded measure of mutual information 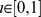:
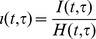
(4)The numerical implementation of Eq. (1) and Eq. (3) is not as trivial as it may seem at first glance because the two dimensional joint probability density function in Eq. (2) has to be obtained from small and noisy data-sets. A naive approach considering an equidistant binning of the density function therefore is problematic. In particular sparsely and unequally distributed sample points can lead to in erroneous deviations [49]. In a typical MEG paradigm a data-set consists of about 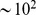 trials. If one includes a moving time average 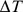 in the range of 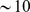 to 50 ms, the total number of samples sums up approximately from 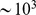 up to 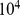 data-points per estimate. Furthermore, a sliding window technique makes the estimate less prone to a jitter of the underlying connectivity across the trials. Such pooling of the sample assumes a quasi-stationary state of the system within the specified time interval. [50] summarize a variety of approaches for the estimation of information measures each holding specific biases or statistical errors. We implemented the estimator by Kraskov and colleagues [2]. They suggested a binless estimator of the entropies such as Eq. (1) and Eq. (3), which is based on a 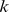-nearest neighbor search. This approach is adaptive on the density of the data-points and exhibits a minimal bias, which makes it more accurate and more stable compared to approaches based on binning [50]. As a rough idea of the algorithm, the search of the neighbors can be implemented very efficiently with help of a ranking of a distance vector. The distance to the 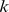-th neighbor is obtained by counting the ranked vector. This is computed for each of the data-points and averaged for the estimation of the entropy [2]. In our approach we include periodic boundary conditions for the cyclic variables. We chose the number of neighbors following the suggestions of [2]. They found that the precision of the estimator depends on the ratio of the neighbor parameter 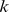 and the total number of samples 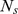. Thus, we set the number of neighbors adaptively to the input with 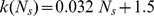. To assure a good performance in speed the core of the algorithm is implemented in C-language and integrated in a MATLAB environment.

The information about the synchronicity of two cortical areas is represented by a connectivity pattern. The driver is defined with the index 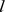 at the time 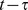 and the responding with 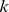 at the time 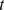. Each pattern forms a two dimensional map, which contains the strength of the phase synchronization Eq. (4) as a function of the time 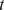 on the abscissa and the time-delay 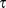 on the ordinate. A connection between two sources is represented by a cluster of an increased synchronization within a pattern. The extension of such clusters provides information of the persistence and the height of the cluster indicates the strength of a connection. Several factors, which might have an influence on the appearance of connectivity clusters are discussed in the Results.

To complement our connectivity analysis we suggest a statistical evaluation based on the false discovery rate (FDR) by [42] as a rating of significantly increased connectivity values within a pattern. The method is adaptive to the data and easy to implement. It is well-known in many fields such as verifying significant voxel in fMRI [41] or testing significance in connectivity analysis of EEG and MEG data [28], [51]. The significance threshold is determined with 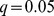 denoting the ratio of false active to active values using pre-stimulus data. We assess the distribution of the null hypothesis – roughly spoken the unsynchronous or unconnected state – with the help of a pre-stimulus segment from 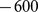 to 0 ms of the pattern. 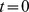 ms represents the stimulus onset.

### A modified Rössler network

In this part the model is presented, which is used to generate synthetic data-sets for testing and refining our methods. Our aim is to detect interrelations among cortical populations which are reconstructed from measured electromagnetic fields outside the head. However, we are primarily not interested in setting up physiologically realistic models such as realistic and complex neuronal mass models, which play an important role in an explicit and realistic modeling of cortical oscillations [52]–[54] because we will apply the method to real MEG measurements (Results: Application to MEG data). Instead, for our demands a good controllability and high simplicity is of great importance because our proposed method in detecting synchronicity is of universal character and therefore ideally independent of a chosen model. In [3] we already proposed the Rössler oscillator [55] as a basis in generating specific data-sets. Coupled Rössler oscillators form a well-explored standard system in the context of synchronization and feature a complex dynamics controlled by just a few parameters [13], [20], [31], [33], [36]. Our system is given by mutual coupled non-autonomous stochastic ordinary differential equations of third order:
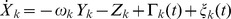


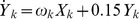
(5)

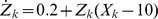
with 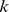 indexing the oscillator number and the intrinsic uncorrelated Gaussian white noise 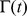 of unit variance. As a prevention of resonance within the network the cyclic frequency 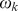 is Gaussian distributed with a mean of 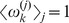 and a standard deviation of 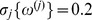. It is drawn for every trial under the side condition that the frequency is positive. We interconnect 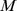 oscillators with help of a linear amplitude coupling 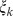, which also considers time-dependent connections with arbitrary temporal delays.
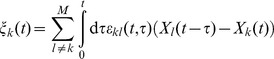
(6)

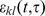
 denotes the time and delay dependent coupling strength between the driver 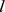 and response 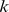. The iteration of Eq. (5) is done using a Runge-Kutta method of fourth order with a step size of 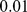 and a resampling of every 30th step. We use a randomized initial condition near the steady state trajectories and neglect the first 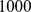 iterations of transient dynamics. The computed time-series of the 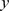-components given by Eq. (5) serves as the signal amplitudes in our further investigations. We generate time-series with a length of 1200 ms each and sample the data with a frequency of 600 Hz as in typical MEG recordings.

**Figure 1:**
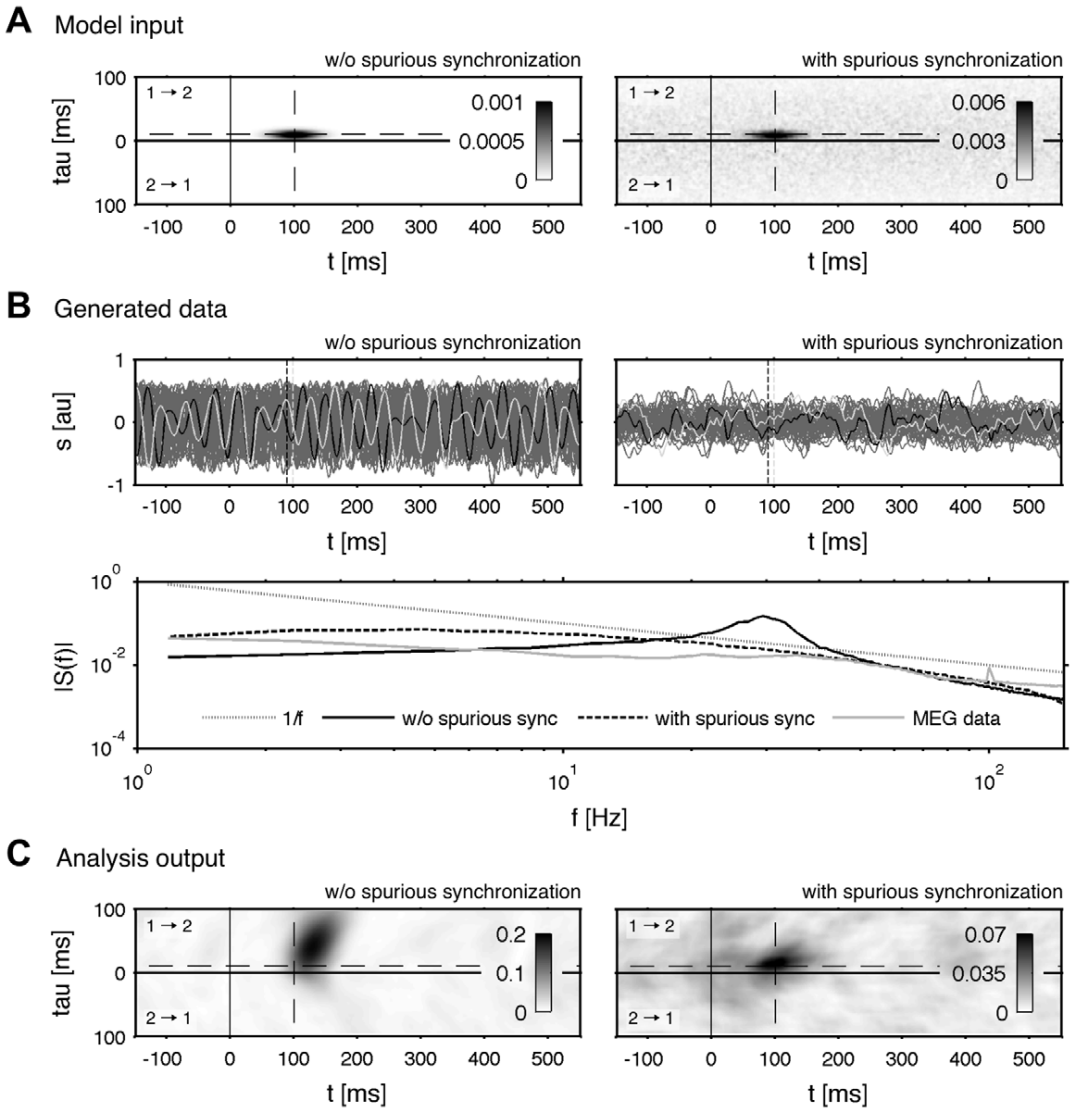
Properties of the synthesized data. The synthetic data were generated in Eq. (5) by linear amplitude coupled Rössler oscillators. **A** Linear coupling strength 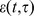 as the input of Eq.(5) for modeling a connective structure. The pattern on the left is given by a Gaussian in the 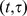-plane centred at 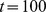 ms and 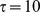 ms. The pattern on the right includes an additional spurious non-zero background activation (right), which is generated by Gaussian filtered Poisson noise and decays for high time-delays. **B** Simulated time-series 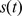 with oscillatory (left) and a more stochastic (right) behavior. The maximum of the connectivity is shown via dashed lines: black indicates the driving system and grey the driven system. A comparison of the corresponding power-spectra 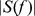 points out a 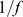 characteristic (dotted black line) for the system including a spurious background synchronization (dashed black line), which can be also observed in MEG recordings (grey line). **C** Corresponding mutual phase information 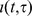 for 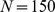 trials. A high connectivity is indicated by a high mutual information. The system featuring a background synchronicity (right) holds a damped, less extended and weaker connectivity in the 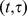-plane.

Two dimensional Gaussians with a standard deviation of 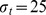 ms and 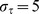 ms are arranged in 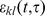 of Eq. (6) as specific connections. A simple linear chain is modeled in a 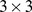 network. Fig. 1A shows the coupling strength between oscillator 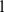 and 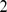 (in the top half) and between 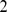 and 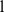 (in the bottom half). Until now it is still not clear what mechanisms determine the amount of the delay time between two cortical areas. It seems to be that the phenomenal delay between two areas is much shorter than physiological axonal conduction time. e.g. Roelfsema et al. found a small time-lag of 2 ms in a visuomotor integration study using cross-correlations [56], whereas Tallon-Baudry et al. measured larger time-lags of 5.4 ms and 12.4 ms in visual short-term memory study applying the phase coherence [57]. A delay of 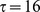 ms was reported in the context of a connectivity analysis by Vicente et al. in a motor task [28] and also by Hinrichs et al. in a visual spatial attention task [58]. In our toy model we use a constant delay of 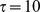 ms as a rough choice, which is in the range of observable time-lags.

With our model we generated two types of data which differ in their spectral properties. The first type is a conventional Rössler oscillator with a frequency peak at approximately 30 Hz, cf. the spectrum in Fig. 1B. For the second type a spurious background synchronization is added heuristically. This is to emulate the ubiquitous correlated brain noise. This background noise is added in the coupling fields 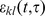. We choose a filtered spatial Poisson noise (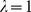) in the 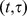-plane to include temporal correlations in the coupling and to generate smooth transitions among two steps. The filtering is done with a Gaussian kernel (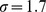 ms) and results in a normally distributed spatial correlated noise pattern. Additionally, the spurious couplings to higher delays is damped using a Gaussian envelop on the 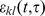 pattern with standard deviation 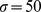 ms. The right pattern in Fig. 1A shows the input of our modified Rössler system Eq. (5) including an additional Gaussian connection, which generates a numerical stable dynamics. The noise level is 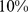 of the maximal strength of the Gaussian shaped connectivity. The generation of the random background activation is repeated in every trial. In contrast to a conventional Rössler system such a modification of the system results to a broad-banded spectral behavior, which shows in good approximation a 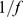 characteristic (Fig. 1B). The change of the spectral behavior is a result of the collapsed Rössler attractor due to the delayed feedback with a noise driven dynamics in the 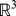. A spectral 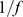 phenomenon is typical for electrophysiological recordings [25], [37], [38].

Thus, we have developed two simple models, which serve as useful and controllable tools. They are not supposed to create a realistic physiology, but rather they support complementary tastes regarding their dynamics: the system based on the conventional Rössler oscillator provides a complex oscillatory dynamics and in contrast the modified one a stochastic 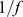 dynamics, cf. Fig. 1B.

## Results

This Section is divided into three main parts with regard to their content. In the first part the mutual information of the phase 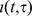 is checked on its reliability when used on correlated and noise contaminated data. We introduce a simple but efficient method in removing correlations and compare our approach to an alternative based on an ICA. Further, we address the stability of 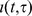 regarding the total number of trials and compare 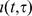 to the cross-correlation, phase coherence and the phase synchronization. In the second part MEG data-sets are simulated and the compatibility with the mutual information of the phase is verified. The third part shows the result of our approach applied on an MEG study of Steinberg and colleagues [32], who investigated the processing of conditioned face stimuli and found an early change in activation in the frontal and temporal region. We are able to support their result by providing evidence of an increased phase synchronization between both areas.

### Verification on synthetic data

#### Temporal correlation of the connectivity

A connection between two sources manifests in an extended cluster of increased synchronization within a connectivity pattern. The cluster extension depends on the underlying coupling of the processes and is also influenced by a correlation in 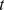 and 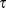 direction [3]. The origin for temporal correlation of the synchronicity are twofold: first, it is caused by an intrinsic inertia of the process. A physical system cannot switch instantaneously from an unsynchronized into a synchronized state [20]. Second, pooling the sample within a time window 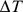 marks a crucial step to reduce noise induced effects in the mutual information estimator. However, as a side-effect the connectivity may be smoothed and therefore may be correlated within the pattern. This effect is negligible as long as the chosen window size is small compared to the intrinsic mechanisms and one is interested in cluster sizes or time scales larger than 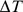, respectively [3]. To speed up the computation time for the calculation of a complete connectivity pattern we do not evaluate the mutual information on the maximal temporal resolution given by the sampling frequency of 600 Hz. Because of the correlative effect after pooling the data within a certain time window 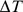, it is sufficient to compute 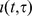 on a coarser sampled triangular grid in the 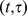-plane. We choose a distance of 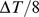 between two neighbored connectivity estimates. With 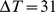 bins (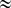
50 ms) the computation time is increased rapidly without loosing information due to the coarse sampling technique.

In Fig. 1C the detected functional connectivity 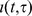 among two sources is depicted. On the left the computed 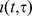 of a conventional Rössler system is shown and on the right of our proposed system with a spurious background coupling (cf. Methods). Both data-sets are created by applying the coupling strength 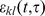 of Fig. 1A as input to Eq. (5) and Eq. (6). The synchronized regime of the unmodified oscillatory system is more pronounced in terms of the strength and the temporal extension compared to the modified stochastic one. The reason is that in the modified Rössler network – due to the break down of the limit cycle – a high noise input desynchronizes both system. In the conventional Rössler system the oscillations are less damped, so that both systems diverge slowly. This leads in contrary to the modified Rössler system to a shift in the connectivity pattern, i.e. the bias of onset and delay of a detected connectivity depends strongly on the underlying dynamics.

#### Linear mixtures and additive noise

**Figure 2:**
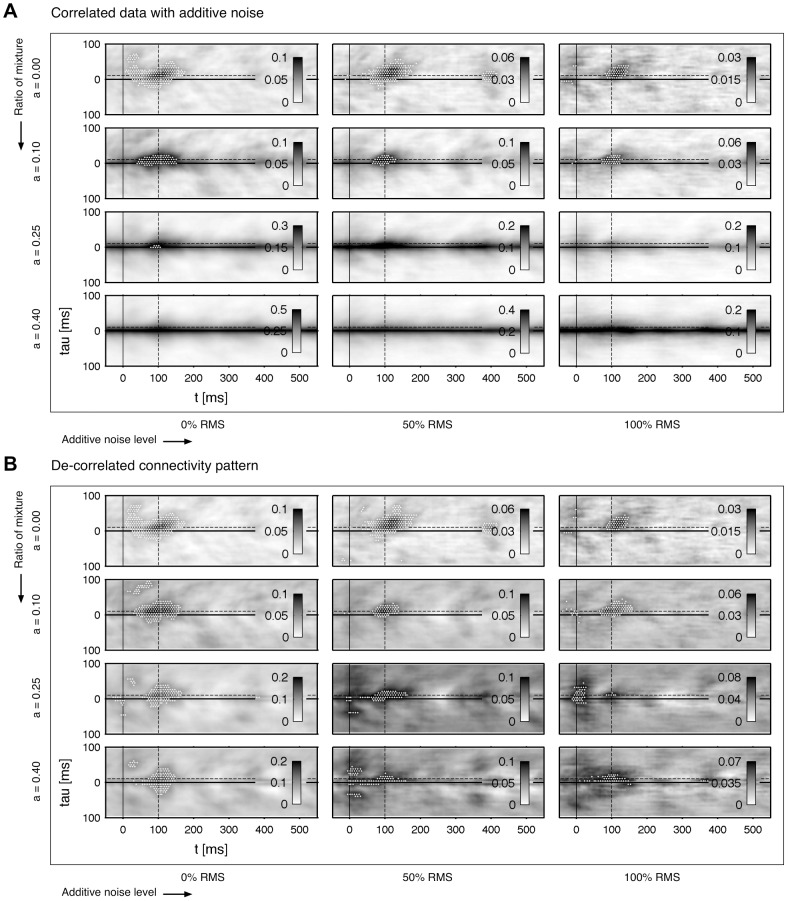
Linear correlated data with additive noise: pre-stimulus based de-correlation. The underlying bivariate data-set consists of 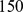 trials with a 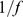 spectrum, cf. Methods (A modified Rössler network). **A** 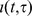 of two mixed sources. Each pattern represents a connection directed from 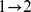 (upper) and 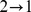 (lower part). A sliding window 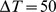 ms is applied for the estimation. The dashed line indicates the modeled connectivity, cf. Fig. 1A. Parameter 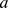 sets the mixing strength as referred to Eq. (7). In **B** the pattern is de-correlated by subtracting Eq. (8), which is fitted in the pre-stimulus interval from 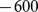 ms to 0 ms. The 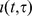 is computed on a triangular grid with a distance of 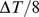 between neighbored estimates. Significant increased synchronization is indicated by a white dot on the grid using a FDR with 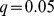.

The investigation of phase synchronization in human EEG or MEG data is ambitious. In general, the investigator may be interested in causal or correlative relationships among brain areas, which are caused by specific underlying mechanisms of the brain. Directed effects are usually termed as effective connectivity and correlations as functional connectivity [47]. However, reconstructed sources typically show artificial correlations [19], [59]. In sensor space the raw data represent a linear mixture of the underlying sources. Inverse techniques are supposed to map the data with help of head-models in the cortical space by a sophisticated separation of the channels Many external factors influence the quality of the source reconstruction procedure: measurement noise, signal degradation through amplifying and filtering, limitations of the head-model or artifacts (such as muscle activity, breathing or eye blinks, to name a few), which results in an imperfect reconstruction with partially correlated sources. Therefore, we want to introduce a simple method, which efficiently removes the influence of an incomplete source separation. Further, we want to assess the performance of our proposed de-correlation and compare it to an approach based on the independent component analysis (ICA) [40]. The instantaneous mixture of two time-series 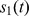 and 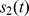 is given by:
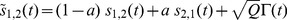
(7)The parameter 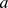 controls the symmetry of the mixing and 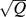 is the level of the additive Gaussian white noise 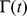 with unit variance. The level is set implicitly by adjusting it relative to the RMS value of the signal. A choice of 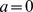 denotes the unmixed case and 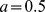 a complete symmetric mixture of both signals. The bivariate data-set consists of 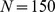 trials of spurious synchronized Rössler oscillators with 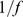 distributed spectra as described in the Methods (A modified Rössler network). The estimation of 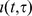 for mixture of both time-series is depicted in Fig. 2A. The estimate is extremely stable to additive noise. Even an amount of 100% RMS is reconstructed with high accuracy. However, the performance is sensitive quickly to the symmetry of the mixture. At a mixture of 50% (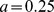) the connectivity is vanished almost completely by the correlated sources. High ratios of mixtures lead to increased correlations at small delay values. One can say that the correlation is in a good approximation a function of the delay and not a function of the time. It reaches its maximum at a zero time-lag and decays with higher time-lags. Strictly speaking, the connectivity which is determined by the coupling of both systems, is covered by the correlation of the mixture.

#### Pattern de-correlation

**Figure 3:**
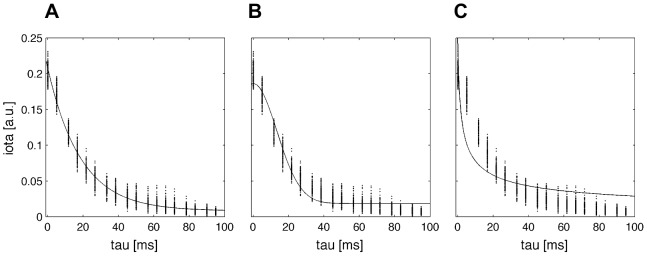
Applied functions for the de-correlation step. Shown are three least mean square fits using the Matlab curve fitting toolbox with the following types: **A** exponential, **B** Gaussian and **C** power law function. The functions were fitted on the pre-stimulus interval of a connectivity pattern (cf. Fig. 2A with a mixture of 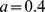 and an additive noise level of 100 RMS).

The basic idea of our approach is to enter the underlying connectivity by simply removing the correlative part on pattern level which is caused by the mixture. By this, we assume that there is no underlying specific connectivity in the pre-stimulus interval. In an experimental design the duration of the stimulus or the inter-stimulus interval is usually randomized. The randomization ensures that the timing of the pre-stimulus interval is jittered with respect to the following stimulus. The jitter also destroys spurious phase relations in the pre-stimulus interval across the trials. First, we need to assess a functional relationship of the delay dependent part. The pre-stimulus interval of 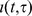 is averaged over the time and projected onto the 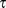 axis. Both coupling directions are processed equally, that means the pre-stimulus interval is additionally averaged over both directions. We find a good functional agreement with an exponential fit model (cf. Fig. 3):
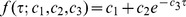
(8)The parameter 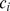 are fitted to the data and subtracted form the whole pattern. Afterwards a statistical rating in form of a FDR control (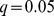) is applied on the data. The result of the de-correlation step are shown in Fig. 2B: the connectivity is recovered even for high mixtures and noise values. The direction, which is given by the symmetry of the cluster, is degraded at extreme ratios (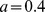). Additionally, the influence of high noise level leads to inaccurate estimates regarding the timing of the connection.

**Figure 4:**
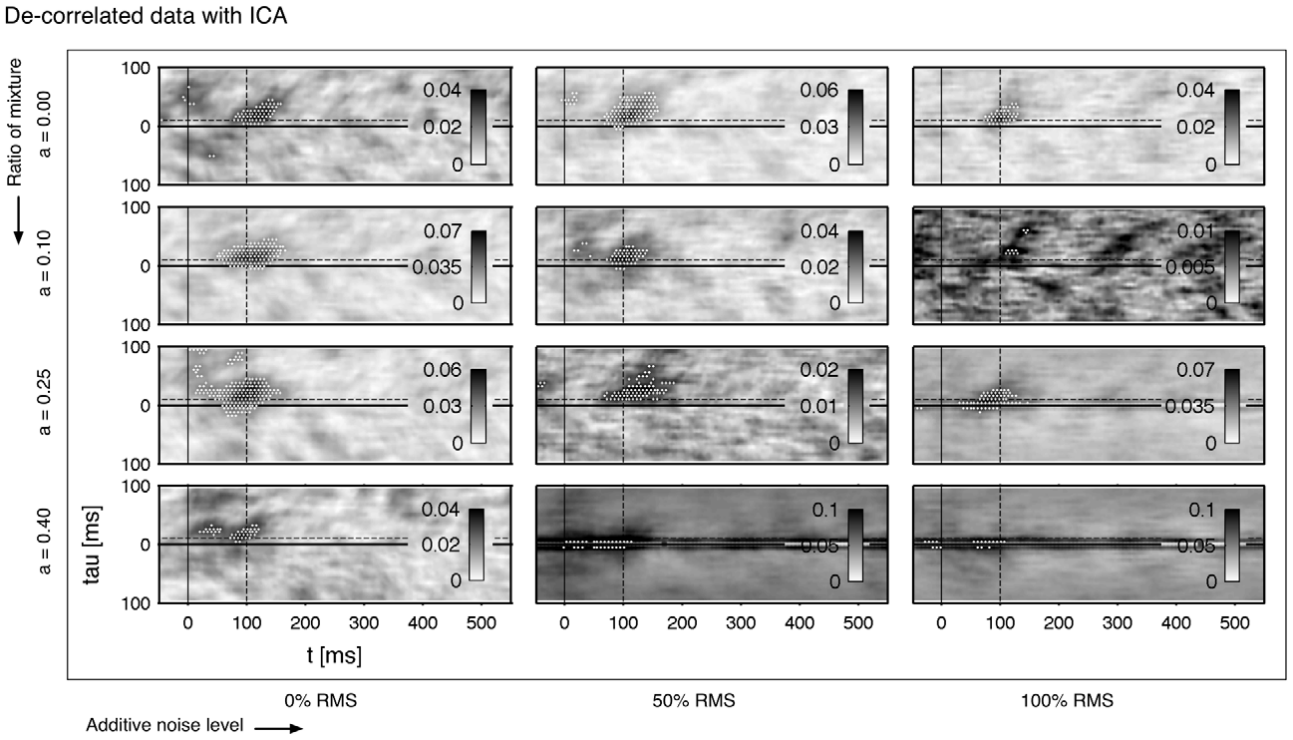
Linear correlated data with additive noise: ICA based de-correlation. In contrast to Fig. 2B the bivariate time-series are directly de-correlated with help of an independent component analysis (ICA) using a Matlab based toolbox of [40]. The resulting patterns 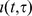 of the de-mixed sources are shown. The same data-set is used as in Fig. 2A. The computation and statistical validation of 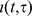 are performed analogously to the results of Fig. 2A.

Next we compare our approach to a direct separation of the signals by using an ICA by [40] (FastICA is a free Matlab toolbox downloadable at http://research.ics.tkk.fi/ica/fastica/). To avoid that indices are mixed up incidentally and therefore the direction is reversed after the ICA step, the cross-correlation is calculated between the sources before and after the separation. A higher cross-correlation assigns a separated source to the corresponding source index. After the separation 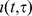 is estimated. The results are shown in Fig. 4. The ICA works reliably for mixed or for noisy data. But the combination of both strong correlation and high additive noise leads to poor results of the reconstructed connectivity. In comparison to our approach the performance is worse for the extreme parameter settings. In addition, in some cases of high noise the fast ICA algorithm fails and the routine is interrupted due to a missing convergence of the solution.

To summarize briefly, we implemented and compared an effective and important de-correlation step on pattern level by fitting a decaying function to the pre-stimulus interval of the pattern. Our approach is in terms of stability more reliable than a source separation with the fast ICA [40], which fails at high noise levels. On the next stage we want to challenge our methods on more realistic test situations. In the Results (Dipole simulation) we consider a set-up including correlated noise, which serves as a basic model for brain or sensor noise after erroneous source reconstructions.

#### Variation of the trial number

The analysis of nonstationary dynamics typically demands a trial based data-set. In a cognitive task the number of trials is usually split into several experimental conditions, which makes the total number of trials very limited. Therefore, we test the connectivity in dependence of the number of trials.

**Figure 5:**
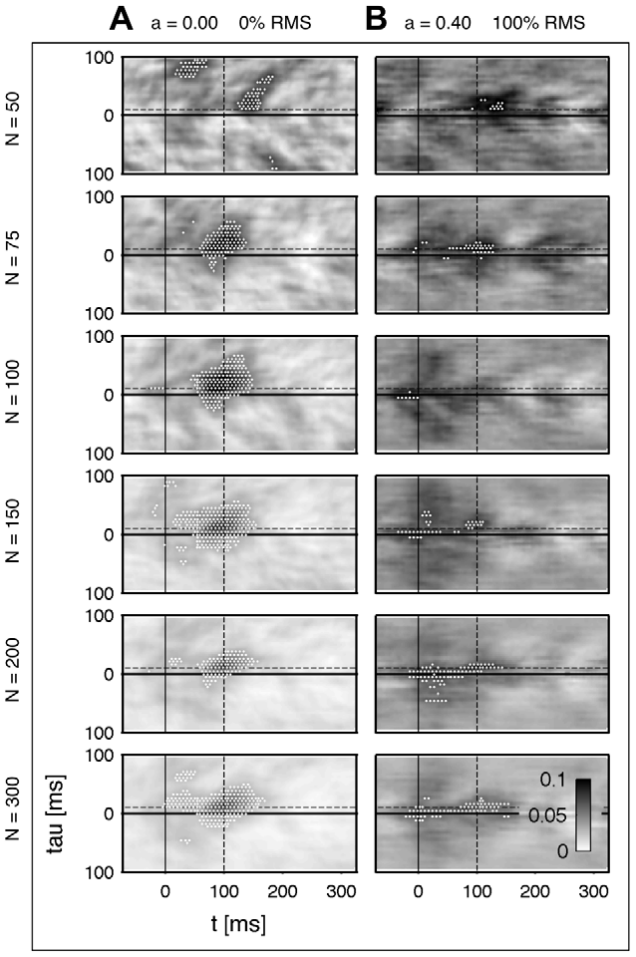
Variation of the total number of trials. Estimated synchronization 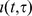 for a variate number of trials 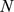. **A** Two uncorrelated and b) two strongly correlated and noisy sources. The same de-correlation step is applied as in Fig. 2B. The same data-set is used as given in Fig. 2. The black dashed line indicates the center of the modeled connectivity.

We used the identical data as in the part before. Fig. 5A shows the result of uncorrelated data without additive noise. The reconstructed example with 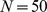 holds high background fluctuations, which results in this case to false positive detection. The patterns are recovered correctly with 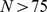 trials. Next, we take the worst case regarding the instantaneous mixture and the noise level. The resulting patterns are given in Fig. 5A. The results are much more unstable as already seen in the last part. Regarding the results of Fig. 5B and the previous results in Fig. 2B a general statement is difficult and depends strongly on the specified system. The connectivity of the modified Rössler system (Fig. 1C, right) is weaker compared to the unmodified Rössler system (Fig. 1C, left) because the intrinsic noise level is relatively higher. However, our impression is that in most cases a trial amount of 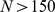 marks a sufficient number which is capable detecting most interactions, although it has some limitations regarding the correct timing. It should be noted that such inaccuracies in the timing are a result of an extreme degradation of the signal, i.e. the applied time-series were mixed with 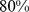 (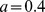) and the noise level was with 100% RMS identical to the signal level.

#### Comparison to alternative phase measures

**Figure 6:**
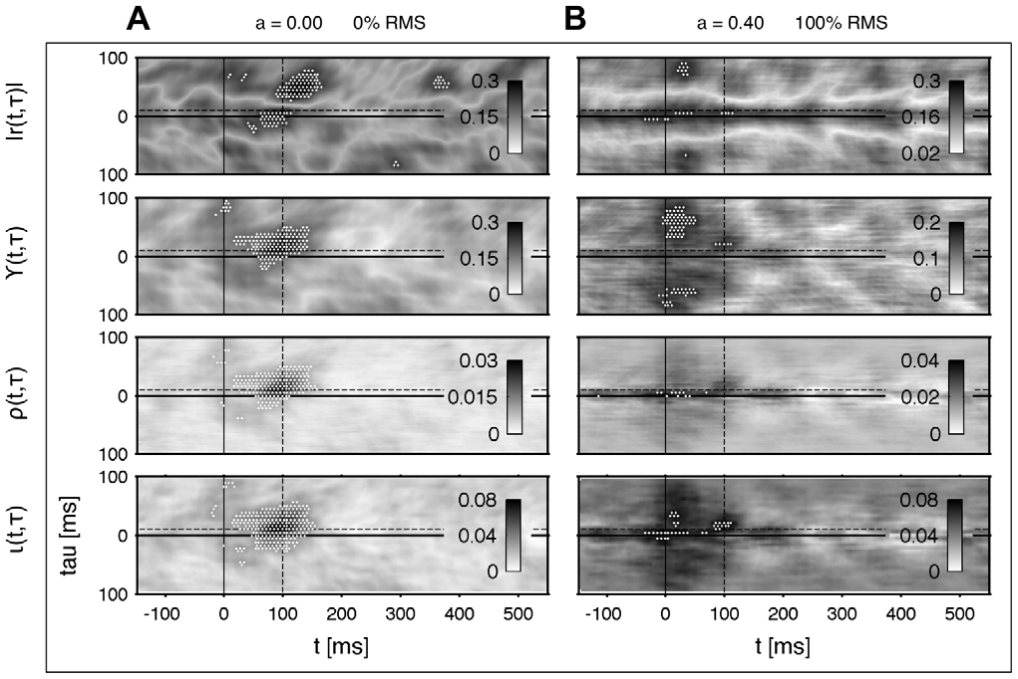
Qualitative comparison to cross-correlation, phase coherence and phase entropy. **A** Unmixed case with 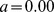 and without additive uncorrelated white noise, **B** strong correlated sources with 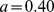 and high additive uncorrelated white noise with 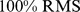. 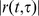 denotes the absolute value of the cross-correlation of the amplitude. 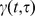 is the phase coherence in time-domain, cf. Eq. (9) with 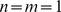, 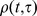 the phase synchronization based on the Shannon-entropy, cf. Eq. (10) with 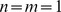, and 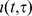 the mutual information of the phase, cf. Eq. (4). The same data-set with 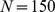 trials is used as shown in Fig. 2A. The connectivity patterns are estimated with a moving time window 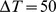 ms and de-correlated analogously to Fig. 2B. The temporal coordinate of the underlying connectivity is indicated by a black dashed line. Because 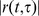, 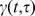 and 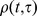 are estimated in every bin, the significant increased correlation is indicated by a white area (FDR with 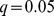).

In [3] we already introduced the concept of connectivity patterns by applying a parametric time-delay, where we combined this with the phase coherence and the synchronization index. Both techniques assess an explicit order n∶m of the synchronization. In the context of a data driven philosophy this might be a disadvantage because of the increasing complexity of the results. Nonlinear measures such as the mutual information reduce the complexity significantly. Especially this property makes them very attractive for the investigation of systems with many degrees of freedom. In this section we want to compare the stability of the mutual information to the cross-correlation of the amplitude 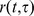, the phase coherence 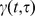 and the synchronization index 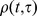. With the phase difference of order 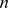 and 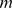 among two sources 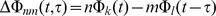 the phase coherence 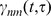 is estimated by
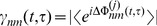
(9)A normalized synchronization index [23] is calculated with the help of the probability density function of the phase differences 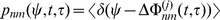 by the following expression:
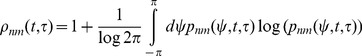
(10)
Fig. 6 shows the results of the estimated functional connectivities. The same data set of 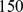 trials is used as in Fig. 2. The patterns are de-correlated following our approach in the Results (Pattern de-correlation). Due to the fact that the underlying data is modeled with a linear amplitude coupling of Eq. (6) – that means both systems synchronize directly – we can choose the indices for the synchronization order of 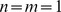 and abbreviate with 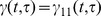 and 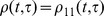. In Fig. 6A is shown that the quality of all phase measures is very similar regarding the significant values of connectivity. For the cross-correlation the connectivity cluster is divided by a zero-crossing due to oscillatory properties of the data. We want to emphasize again that such typical artifacts of amplitude based methods are problematic in our approach leading to an underlying periodic fluctuation [3]. The structure of the underlying fluctuations strongly depends on the spectral properties, which, in turn, are in general time-dependent. In contrast to the phase measures, it is not possible to forecast a simple de-correlation technique as in Eq. (8), which works for time invariant and decaying correlations in 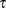 direction. Fig. 6B depicts the results of a degraded data-set. The mixing ratio and noise level is identical to Fig. 5B. In this example the pattern based on the cross-correlation 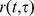 is very noisy. Throughout the pattern the background fluctuation is pronounced and inhomogeneous. Although our de-correlation step might be problematic on amplitude based correlation measures, the FDR discovers two small clusters symmetrically in the delay component. The cluster location is detected correctly regarding the time component but incorrectly regarding the delay direction. The phase coherence 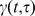 also suffers from high inhomogeneous background noise. Further, it seems not very robust to additive noise because of two significant false positive extensive and symmetrical artifacts. The results of 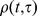 and 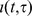 are very similar. In this specific data-set both measures are biased to correlations shifted to the onset. But compared to 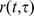 and 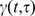 the patterns are homogeneous regarding their background noise. If one wants to restrict on linear interactions with a direct 1∶1 synchronization 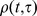 may be used as an alternative to 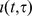. Both are based on an entropy estimate, but in contrast to 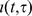 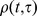 requires just a one dimensional density function. [51] suggests a restriction to linear measures on stationary data. Further, they claim that a higher sensitivity leads to less stability. But our example shows that the results of the 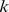-nearest neighbor estimator are comparable in terms of its stability to competing less complex approaches. We even show that the analysis can be expanded to arbitrary processes, which can be nonstationary and even include an arbitrary order of synchronization.

### Dipole simulation

#### A brief introduction to current dipoles and beamforming

In this section we verify, whether the method used for the source reconstruction – focusing on the beamforming technique – is compatible to measures of phase synchronization. This is addressed by a connectivity analysis of data, which is simulated as an MEG recording and subsequently reconstructed again as cortical source activity. In a connectivity analysis one is interested in interdependencies among sources. In MEG simulations point-like sources can be modeled as equivalent current dipoles [39], [60]. We use such dipole-like sources with time-courses from our data-sets as source waveforms to simulate measured data of intracortical activations with a prior defined connective structure. Although more sophisticated models of spatial extended sources exist [61], we want to restrict to point-like sources with dedicated complex dynamics. The cortical sources of the simulated recordings are estimated trial-wise by using a beamforming technique. The beamforming method can be understood as an adaptive spatial filter, which is able to map the recorded sensor data onto the source space as a dipolar source density by the optimization of linear filter weights. For the optimization of the weights the beamformer assumes that sources are not correlated in a linear fashion at the same time [44], [62]–[64].

Our goal is to prove if our approach of assessing functional connectivity by the estimation of underlying connectivity patterns can be reconciled with sources reconstructed by a beamforming technique. We try to model under more realistic conditions by including correlated noise of unsynchronized cortical point-wise dipolar sources in the source space [65], [66] and thermal sensor noise in the sensor space [39]. Both types result in spurious correlations of underlying source activities, which were not addressed so far (cf. Results: Verification on synthetic data). Because we demand source activities reconstructed trial-wise, the source reconstruction cannot be applied as usually done on averaged data. To handle a noise reduction on raw data without the necessity of an average across the trials we want to motivate the use of a linear WMA filter. [31] already showed that phase sensitive measures are in principle compatible, but they did not consider a time and a delay sensitivity in their analysis. Further, they used symmetrically coupled systems and therefore had to restrict to short pre-stimulus intervals in the beamformer estimation.

#### Simulation of synthetic MEG recordings

The source signals are given by time-courses generated by a coupled Rössler system as described in the Methods (A modified Rössler network). A pair of oscillators is connected by a linear amplitude coupling given in Eq. (6). We use both the conventional and the modified Rössler system as the underlying data of sources with a known connective structure. The unmodified system holds a more oscillatory dynamics with a distinct spectral peak at 30 Hz (cf. left panel of Fig. 1). In contrast, the modified system shows a broad-banded 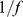 distribution in the spectrum (cf. right panel of Fig. 1). We simulate a trial-based MEG data-set, which consists of 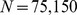 or 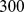 epochs. Each epoch lasts for 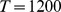 ms with a sampling frequency of 600 Hz including a 600 ms pre-stimulus interval. The connective setup consists of three dipoles, which are placed in the calcarine sulcus (V1, dipole 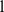), extrastriate body area (EBA, dipole 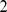) and in the superior temporal sulcus (STS, dipole 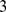) all of which respond to visual stimuli. The dipoles are arranged in a linear unidirectional chain as given in Table 1. The coupling strength 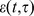 induces a time dependent and delayed synchronization of the dipoles, cf. Table 2. The locations of the three dipoles are fixed during all simulations. Their orientation is tangential which means that the dipole is approximately orthogonal to the surface normal with the shortest distance to the dipole. The tangential direction is chosen randomly for each dipole and each simulation, i.e. it stays constant during the epochs within a specific simulation.

**Table 1:** Linear dipole chain.

**1**	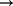	**2**
90 ms	10 ms	100 ms
**2**	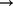	**3**
120 ms	10 ms	130 ms

Schematic illustration of the model of a linear chain used in the dipole simulations. The dipoles are placed in the cortex with fixed coordinates (located in the calcarine sulcus, the extrastriate body area (EBA) and the superior temporal sulcus (STS)) and a randomized tangential orientation, which is held constant for each simulation. The time-dependent amplitudes of the dipoles are given by the time-course of a 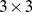 Rössler system.

**Table 2:** Distance and timing of the dipoles.

Driver	Responder	Distance [cm]	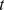 [ms]	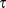 [ms]	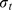 [ms]	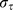 [ms]
1	2	5.5	100	10	25	5
2	3	8.1	120	10	25	5
3	1	5.8	–	–	–	–

Table caption Euclidian distance between each dipole combination and the temporal setup of the connectivities. The coupling strength is given by identical two dimensional Gaussians in the 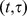-plane similar to Fig. 1A. The location of each Gaussian is set by 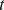, 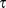 and its temporal extension by the standard deviation 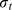 and 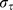. All time-series used for simulations last from 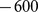 to 600 ms and include a pre- and a post-stimulus interval of equal length.

We consider two noise sources in our simulations: As a first side effect brain noise is implemented by 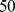 uncorrelated disturbing dipoles with a 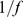 spectrum, which are placed in random fashion within the grey matter. Their location, tangential orientation and time-courses are changed randomly in every trial. A single time-course is unique in each of the simulated recordings and is drawn from a pool of 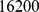 simulated time-series modeled without an underlying temporal defined connectivity. The amplitude of the brain noise is set to constant 10% RMS compared to the three connected sources. To model a variable amount of non stimulus-locked disturbing sources each time-course of the disturbing dipoles is multiplied by a Gaussian window function. To vary the timing the mean value of the Gaussian is chosen from a uniform distribution in 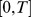. However, the interval of each of the disturbing activations is held constant for simplicity, i.e. standard deviation is set to fixed 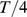. As a second influence thermal noise is modeled by additive uncorrelated Gaussian noise in the sensor space [39].

#### Reconstruction of sources

The sources are reconstructed by a Linearly-Constrained Minimum Variance (LCMV) beamforming [44] with a 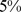 regularization for a spatial smoother and more stable solution [30], [59]. The LCMV beamforming belongs to the class of the vector beamformer, i.e. the reconstructed source is given by a dipole with a specific direction and strength, both dependent in time. A singular value decomposition is applied to reduce the vectorial to scalar response. In dedicated simulations of hippocampal activations, [67] shows that vector beamformer are significantly more stable than scalar beamformer such as the synthetic apperture mapping (SAM). In our simulations the covariance estimation is performed for the complete trial in the time window 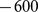 to 600 ms in broad frequency band 1–150 Hz (fourth order bi-directional Butterworth filter) and is necessary for the calculation of the beamformer weights. Both a long time window and a large bandwidth are essential for a stable result and the reduction of biases in the estimation of the covariance matrix [59]. The dipole simulation and source reconstruction are implemented in the Matlab based FieldTrip toolbox for EEG/MEG-analysis [43] (Donders Institute, University of Nijmegen, the Netherlands, the toolbox is downloadable at http://fieldtrip.fcdonders.nl/). The simulated data-set is based on a 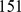-channel CTF system. A multisphere head-model is used in the beamforming procedure [68]. It is generated with the help of a segmented structural MRI file of Fieldtrip's online tutorial data-set *Subject01*. The segmentation of the anatomical MRI data is applied with help of the SPM toolbox [69] (downloadable at http://www.fil.ion.ucl.ac.uk/spm/).

In addition to the internal dynamics, one has to consider additive thermal noise caused by the MEG device [39], which is in a good approximation uncorrelated in time and space. Because uncorrelated noise is not suppressed by the beamformer effortless [59], [70], we suggest the application of a weighted moving average (WMA) filter step with the purpose to attenuate the amount of additive thermal noise in the sensor data by averaging sensor data in a moving time window. In particular, we customized a linear WMA filter composed by a set of different window lengths 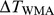. Briefly, the main idea was find a trade off between a sufficient noise suppression while not to deteriorate the signal too severely by choosing exponential spaced time windows 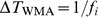 with a corresponding frequency 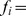
2, 4, 6, 8, 10, 12, 15, 19, 23.5, 29, 35.5, 43, 52, 63, 76, 91.5, 110 and 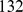 Hz. The smoothing procedure of the time-series is done for each window size 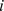 independently and averaged over the complete set afterwards.

To reconstruct the simulated cortical sources we run the following sequence: First, in a pre-processing step we filter the raw data with a linear WMA filter before the beamformer is applied. This marks a crucial step to improve the quality of the reconstructed connectivities. We discriminate two types of sequences for the source reconstruction with the LCMV. In one type, called *WMA+LCMV*, the data is previously filtered with the WMA before the beamformer is applied. Otherwise, the beamformer is used on unfiltered data. In this case we call it *Conventional LCMV*. In the second step the locations of the reconstructed sources are defined. To keep it simple we take the same coordinates as applied for the underlying simulated dipoles. Because the beamforming procedure is spatially smoothed, dislocalizations are not an issue [59], [63], [71]. In the third step the beamformer weights are computed for each of the trials and each predefined location. In the last step the dipolar time-courses are reconstructed by using the estimated weights. Finally, the results are transformed to scalar time-courses followed by the synchronization analysis.

#### Results of the reconstructed connectivities

**Figure 7:**
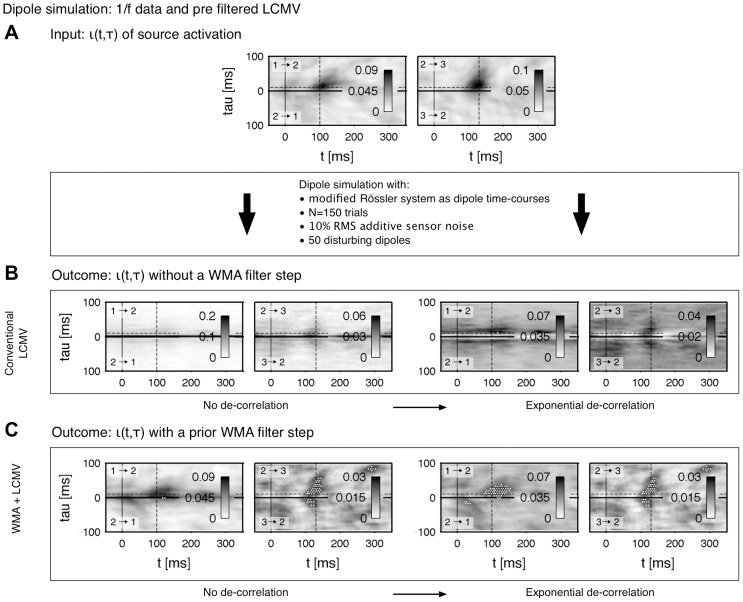
**A** Connectivity 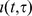 of the input data. Modified Rössler system with a 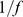 spectrum is used as time-course of three sequentially chained dipoles, cf. Table 1 and 2. 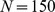 trials were simulated with an additive uncorrelated sensor noise of 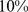 RMS. Brain noise is adapted by 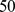 uncorrelated, randomly located and orientated dipoles in the grey matter. **B** Connectivity of the reconstructed source activation with a conventional LCMV beamforming method. **C** Reconstructed connectivity by applying a weighted LCMV in a preprocessing step: data in sensor space is filtered by a linear weighted moving average in order to suppress thermal noise of the MEG device. The smoothed signals are mapped onto the cortex and the functional connectivity among the reconstructed source activation is estimated. In **B** and **C** patterns are unprocessed (left) and processed by an de-correlation on pattern level (right) as suggested in the Results (Pattern de-correlation).

In particular we want to clarify that the functional connectivity in terms of a phase synchronization among sources is preserved after the source reconstruction via beamforming. Because the amount of involved parameters is high and because we are not interested in all side-effects of every parameter combination in detail, we restrict our analysis to the most important parameters. In practice, the amount of trials is set in the experimental design. The outcome of our connectivity analysis is directly linked to the number of analyzed trials. Thus, the variation of the trial numbers forms one aspect in our verification. Further, thermal sensor noise forms a crucial issue in beamforming. The algorithm maps the noise back into to the source space because of its uncorrelated nature. To explore the influence of the thermal noise we vary its strength as a second parameter. As the power of the beamformer in separating sources is limited, correlated brain noise results in a less efficient separation of the sources. The algorithm holds a certain degree of freedom – depending on the amount of data and even on the architecture of the MEG device – to suppress artifacts and separate individual sources properly. With a decreasing number of disturbing influences the performance in separating the sources increases consistently [44], [64], [72]. This results finally in correlations within a connectivity pattern, which grow rapidly with decreasing delays. We addressed a similar issue in the Results (Pattern de-correlation). First, a broad-banded signal of the modified Rössler system is applied as source waveforms (cf. Results: Verification on synthetic data). The dipole simulation comprises 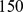 trials with three connected sources, 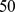 disturbing dipoles and 10% RMS thermal noise. In Fig. 7A the underlying modeled connectivity is shown directly among the time-courses without simulation steps (cf. the right panel of Fig. 1). The patterns in Fig. 7B are based on a source reconstruction via a conventional LCMV method, i.e. without the filtering by a linear WMA in the sensor space. In Fig. 7C the thermal sensor noise is suppressed by the WMA filter. Without any of our proposed corrections the connectivity pattern suffers from strong correlations, which totally cover the underlying connections (cf. left panel of Fig. 7B). By applying the WMA smoothing and the de-correlation of the patterns both of the underlying connections are recovered successfully (cf. right panel of Fig. 7C). If one compares both patterns of the conventional and the filtered LCMV (WMA+LCMV) (Fig. 7B vs. Fig. 7C) and the de-correlation procedure (Fig. 7B and C with left vs. right) the WMA filter seems to be crucial for the reconstruction of connectivity. It also improves the quality of the de-correlation. If two channels are highly correlated, e.g. the connection among 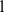 and 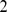 in Fig. 7B, the de-correlation model lacks in accuracy for small lags. Such artifacts disappear when the data are less correlated after the WMA based noise suppression in the preprocessing.

**Figure 8:**
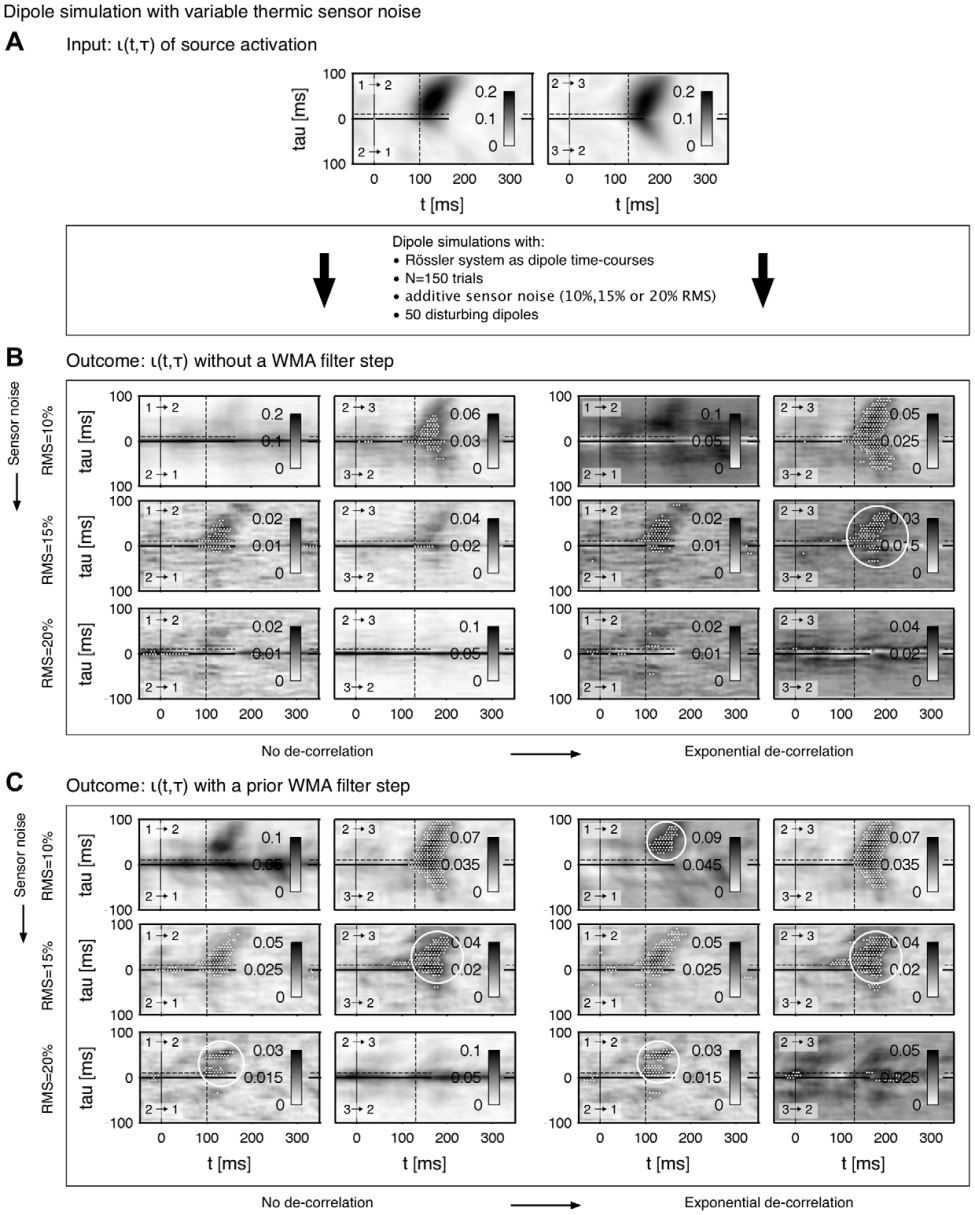
Influence of the additive noise level 10%, 15% and 20% RMS on the reconstruction of the connectivity. **A** Connectivity 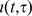 of the input data (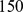 trials of a Rössler system), cf. Table 1 and 2. **B** Connectivity of the reconstructed source activation with a conventional LCMV beamforming. **C** Reconstructed connectivity by applying a WMA filter on sensor data in a preprocessing step (WMA+LCMV). In **B** and **C** the patterns are unprocessed (left) and de-correlated (right) as suggested in the Resutls (Pattern de-correlation). Significant improvements in the connectivity reconstruction compared to the patterns of the conventional beamformer without de-correlation are marked by white solid circles.

**Figure 9:**
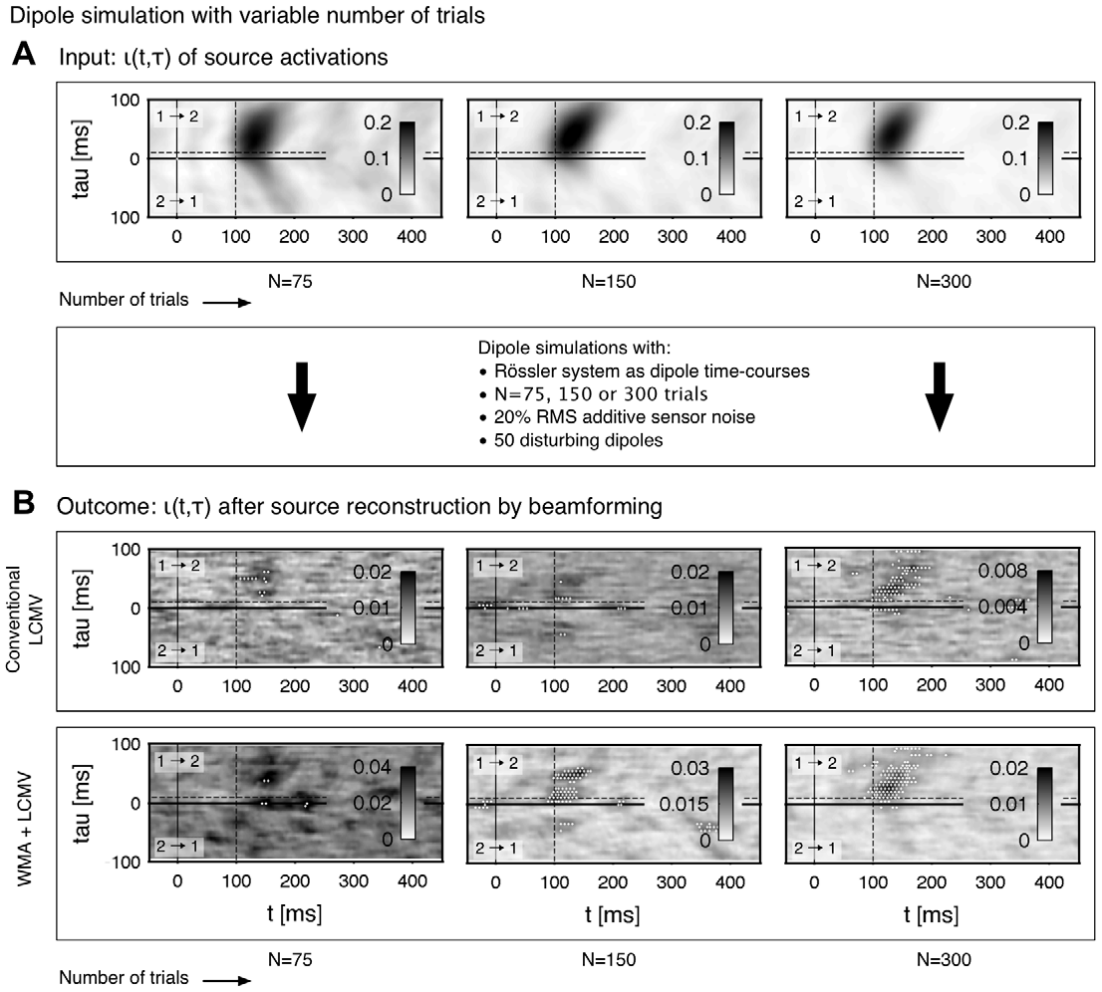
Dipole simulation and reconstruction with Rössler oscillations as source activation with a variable number of 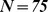 (left), 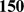 (center) 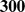 trials (right). The data is simulated with additive uncorrelated sensor noise of 20% RMS and brain noise generated by 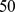 uncorrelated, randomly located and orientated dipoles in the grey matter. **A** Connectivity 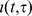 of the connected Rössler oscillators indexed with 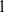 and 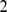, cf. Table 1 and 2. **B** Connectivity of the reconstructed source activation with a conventional LCMV beamforming approach. **C** Reconstructed connectivity by applying a WMA filter to reduce thermal noise captured by the MEG device on sensor data (WMA+LCMV). All patterns in b) and c) are processed by removing exponentially decaying correlations as described in the Results (Pattern de-correlation).

As a next example we switch to time-courses exhibiting an oscillatory dynamics. As already seen in the Results (Temporal correlation of the connectivity) the oscillitary system features a stronger synchronicity than the 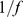 distributed system and is thus better suited for dipole simulation purposes. We first test the robustness to thermal noise which is captured by the MEG device. Basically, thermal noise detected by the instruments is typically smaller than the neuronal signals [39], [66]. Therfore, we tested noise levels at 10%, 15% and 20% RMS compared to the measured signal level. In Fig. 8B and C the results of the simulated and reconstructed connectivity are shown. The estimated patterns of the conventional beamformer procedure recover two out of six possible connections without a subsequent de-correlation (Fig. 8A, left) and three out of six possible connections with a de-correlation step (Fig. 8A, right). The application of the noise suppression in the WMA step increases the amount of the reconstructed patterns on four out of six without de-correlation and five out of six with the de-correlation (cf. Fig. 8C). Last, we vary the number of trials to prove the stability of the functional connectivity in the context of dipole simulations. Fig. 9 shows the analysis outcome for 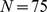, 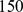 and 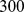 simulated trials. As seen in the results before, the patterns estimated with the weighted beamforming are more reliant compared to the results of the conventional beamforming. In particular, the outcome seems to be satisfactory when 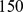 or more trials are used.

To conclude, we showed that our approach in the investigation of phase synchronization is compatible with a source reconstruction based on the beamforming technique. For this purpose we simulated MEG recordings while regarding side effects such as brain noise and thermal noise under the assumptions that orientation, location and the temporal properties of the connections are constant during each simulation. We then applied a LCMV beamformer provided in FieldTrip [43] and customized it slightly to our needs, e.g. we reconstructed single trials and expanded the reconstruction procedure with a WMA noise suppression in the preprocessing. We successfully demonstrated the benefit of our suggested WMA filter in the context of beamformer applications. Although we followed a more qualitative path in examining some few but representative examples, we are able to reveal the benefit of combining diverse approaches in a stringent sequence of analysis.

### Application to MEG data

#### Introduction to the MEG study

After successfully demonstrating the simulation of dipoles based on synthetic data in the Results (Dipole simulation), the method is now applied to a real MEG study on processing of emotionally relevant stimuli comprising the data-sets of 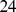 subjects [32]. The purpose is to show that our proposed method also works with real data from complex experimental paradigms and with data-sets which are non optimal regarding the requirements for the estimation of synchronization, such as data-sets with spatially averaged sources and a low number of trials. [32] conditioned neutral faces with aversive odor 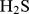 (hydrogen sulfide, unconditioned stimulus) labeled as *Negative*. In a *Neutral* control condition faces were paired with odorless 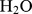 (humid clean air, control stimulus). The stimulus set consisted of 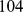 individual faces with a neutral facial expression (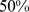 male and 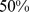 female). Each individual face was shown from a lateral view (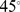, matched in left and right) and from a frontal view. Thus, the conditioned stimuli consisted of 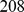 pictures in total. The conditioning procedure was divided into three phases: pre-training, conditioning and post-training. During the pre-training phase (denoted as *Pre*) an MEG was recorded while the participants passively viewed the stimuli. All stimuli were presented twice and in randomized order, i.e. participants viewed 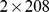 faces. In the conditioning phase 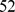 (i.e. 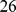 frontal and 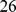 lateral) faces were paired twice with humid air and another 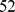 were paired twice with hydrogen sulfide. The post-training phase (denoted as *Post*), which followed the conditioning phase, was identical to the *Pre* phase. A more detailed description of the experimental paradigm can be found in [32]. Steinberg and colleagues [32] hypothesized that distributed affective networks including the amygdala or the orbifrontal cortex would be activated in processing of emotional stimuli earlier than 120 ms after stimulus onset. They found a significant change of the source power in frontal and occipito-temporal regions at early 50–80 ms and at 130–190 ms, and postulated an early functional modulation of occipito-temporal regions by frontal areas [32]. As a possible explanation for this rapid modulation they suppose three possible mechanisms: first, a rapid modulation by the amygdala utilizing direct projections from the sensory thalamus, second, involvement of fast geniculo-cortico-cortical pathways as found in macaque and humans (less than 50 ms), or third, a bypass of V1 involving the superior colliculus and the posterior visual thalamus (extrageniculostriate pathway) as observed in some blindsight patients.

Our aim is to reproduce their result, that is to find evidence for an early directed connection between frontal and temporal regions, while emotionally relevant stimuli are processed.

#### Ethics statement

The study of [32] was performed in accordance with the Declaration of Helsinki, and approved by the Ethics Committee of the University of Münster. All subjects gave their written informed consent prior to their participation.

#### Methods in the MEG study

Starting point is a trial-based spatially integrated minimum-norm estimate (L2-MNE, see [73]) of the frontal and the occipito-parieto-temporal regions on the cortex of each subject. [32] preferred a minimum-norm reconstruction to a beamforming approach because they used a group average. A minimum-norm estimation allows a superficial, spatially more smoothed reconstruction of dipolar activations without a prior knowledge of the underlying dipole configuration [73], [74]. Data for lateral and frontal view of the faces were pooled to gain more statistical power and afterwards bandpass filtered at 3–150 Hz (fourth order bi-directional Butterworth filter) to exclude low frequent artifacts and to attenuate activations of the Delta band. The pooled data-set consisted in average of 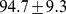 trials (maximum of 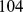 trials) and 480 time points, i.e. 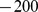 to 600 ms with stimulus onset at 0 ms and a sampling frequency of 600 Hz. We had to exclude six subjects from our analysis because of strong artifacts, especially in the initial pre-stimulus interval, which is crucial for the FDR statistic. These might be a consequence of the pre-processing, which includes many steps, such as artifact rejection, de-trending, filtering and the inverse mapping to source space, for details see [32].

#### Results of the MEG study

**Figure 10:**
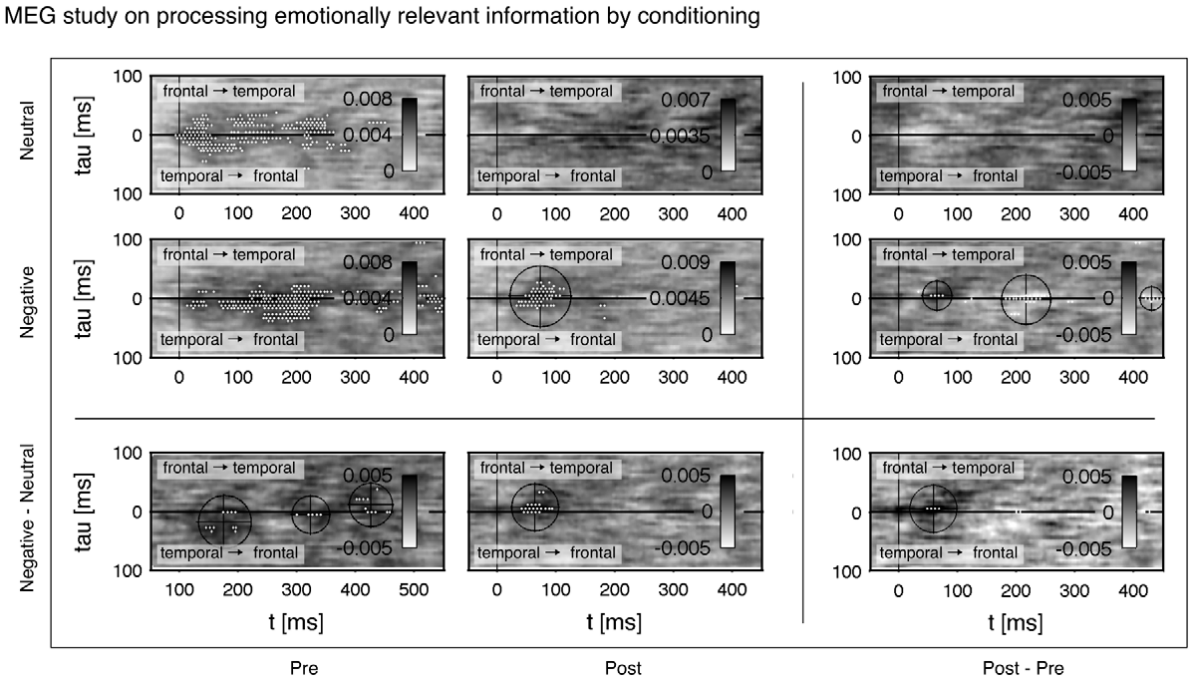
Data from the MEG study of [32] in processing of olfactorily conditioned faces. Shown is 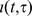 for 3–150 Hz in terms of the functional connectivity between a temporal and frontal cortical area during a passive viewing of faces with neutral expression. The interval 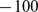
–0 ms was taken to estimate a statistical threshold with a FDR ratio of 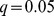. Significant changes are marked by white dots. The patterns were de-correlated with 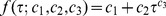. The rows are given by a *Negative* conditioning with 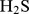 and a *Neutral* control with 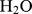. In the contrast *Negative-Neutral* the processing of faces is compensated by subtracting the *Neutral* baseline. The columns denote connectivity before conditioning (*Pre*), after conditioning (*Post*) and the contrast *Post-Pre*, which shows the change in connectivity through the conditioning process. Cluster locations are calculated by the center of gravity and marked by cross hairs. Numerical values of the location are listed in Table 3.

In the following we want to discuss the outcome of our analysis depicted in Fig. 10. Shown is the averaged connectivity 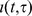 across the 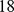 subjects between a temporal and frontal region before (*Pre*) and after the conditioning process (*Post*). 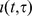 was estimated across the trials including a moving average of 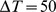 ms. The thresholding was done with a FDR ratio of 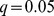. In patterns with contrasts a two-sided FDR was applied. Thereby, contrasts were computed by subtracting the corresponding connectivity patterns. The pre-stimulus interval from 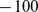 ms to 0 ms was taken as a baseline. Values below 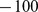 ms were truncated to avoid influence of boundary effects in the statistical thresholding. In some connectivity patterns there are black cross-hairs indicating the center of mass in a cluster. The numerical results are listed in Table 3.

**Table 3:** Connectivity in processing of conditioned stimuli.

	*Pre*		*Post*		*Post - Pre*	
	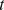 [ms]	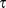 [ms]	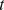 [ms]	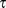 [ms]	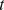 [ms]	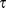 [ms]
*Neutral*	extended		no		no	
*Negative*	extended		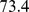	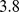	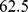	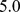
*Negative*					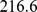	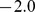
*Negative - Neutral*	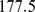	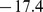	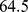	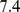	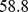	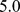
*Negative - Neutral*	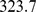	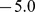				
*Negative - Neutral*	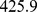	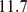				

Numerical values of some cluster locations shown in Fig. 10. Each coordinate 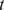 and 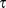 specifies the centroid of mass of the corresponding significant cluster. The time 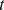 denotes the time after stimulus onset. Here, the directivity is expressed by the sign of the delay time: 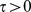 describes a connectivity from frontal to temporal and 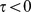 from temporal to frontal. The conditions *Pre*, *Post*, *Neutral*, *Negative* and the corresponding contrasts are arranged in the same way as the patterns of connectivity displayed in Fig. 10.

By taking the contrast *Post-Pre* the direct change of connectivity through the conditioning is uncovered (cf. rows of Fig. 10). However, the subjects perceived each stimulus multiple times, i.e. in this contrast mechanisms of adaptation and fatigue are not respected at all. The adaption in perception to the stimulus is visualized clearly between *Pre Neutral* and *Post Neutral*: in *Post Neutral* the connectivity is lowered and lost significance entirely compared to *Pre Neutral*. Analogously, the *Pre Negative* condition lost its temporarily extended significance after the conditioning phase except for a distinct cluster located at 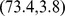 ms in *Post Negative*. This cluster demonstrates the early emotional modulation indirectly, because it even survived the conditioning process despite of fatigue and demotivation. The *Post-Pre Neutral* pattern shows a slight, but non-significant decrease of connectivity in the range of approximately 0–150 ms. This decrease can be regarded as an effect of fatigue and demotivation. Looking at *Post-Pre Negative* there remain two significant clusters with an increased and decreased connectivity, confirming the main results of [32]. Owing to the still included adaptation effect and background noise the size of the significant region is rather small.

The contrast between *Negative-Neutral* takes effects such as fatigue, demotivation or adaptation into account (cf. columns of Fig. 10). Bearing in mind that the underlying activation is generated by different sets of faces, this holds for the assumption that the degree of adaptation has to be equivalent in the *Neutral* and *Negative* manipulation. *Pre Negative* and *Pre Neutral* have both extended connectivity in the post-stimulus interval up to 300 ms. In both patterns one expects an identical activation because of an equivalent stimulus processing before the manipulation phase. So, for the contrast *Pre Negative-Neutral* one would expect no significant cluster. However, this contrast still shows some distributed and weak significant changes in connectivity probably caused by a too high noise level. The pattern in *Post Negative-Neutral* illustrates the change in connectivity regarding the *Neutral* baseline connectivity. Within this contrast we can clearly reproduce the important finding of [32], that there is an early significant increased connectivity between frontal and temporal regions after the conditioning phase. Here, a locally defined cluster emerges within the pattern at 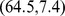 ms as listed in Table 3.

In the last step a meta-contrast, i.e. a contrast of two contrasts, was estimated by computing the differences *Post Negative - Pre Negative - (Post Neutral - Pre Neutral)*. Both, the direct change of connectivity due to a manipulation with hydro sulfide by (*Pre-Post*) and fatigue during the manipulation (*Negative-Neutral*) are considered in this contrast. Within this pattern a pronounced increase emerges in the post stimulus up to 100 ms between frontal and temporal areas. Further, a weak barely significant decrease is located at about 200 ms from temporal to frontal direction. Unfortunately, the background noise level is increased by generating two contrasts in such way that the significant cluster located at 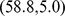 ms is small.

**Figure 11:**
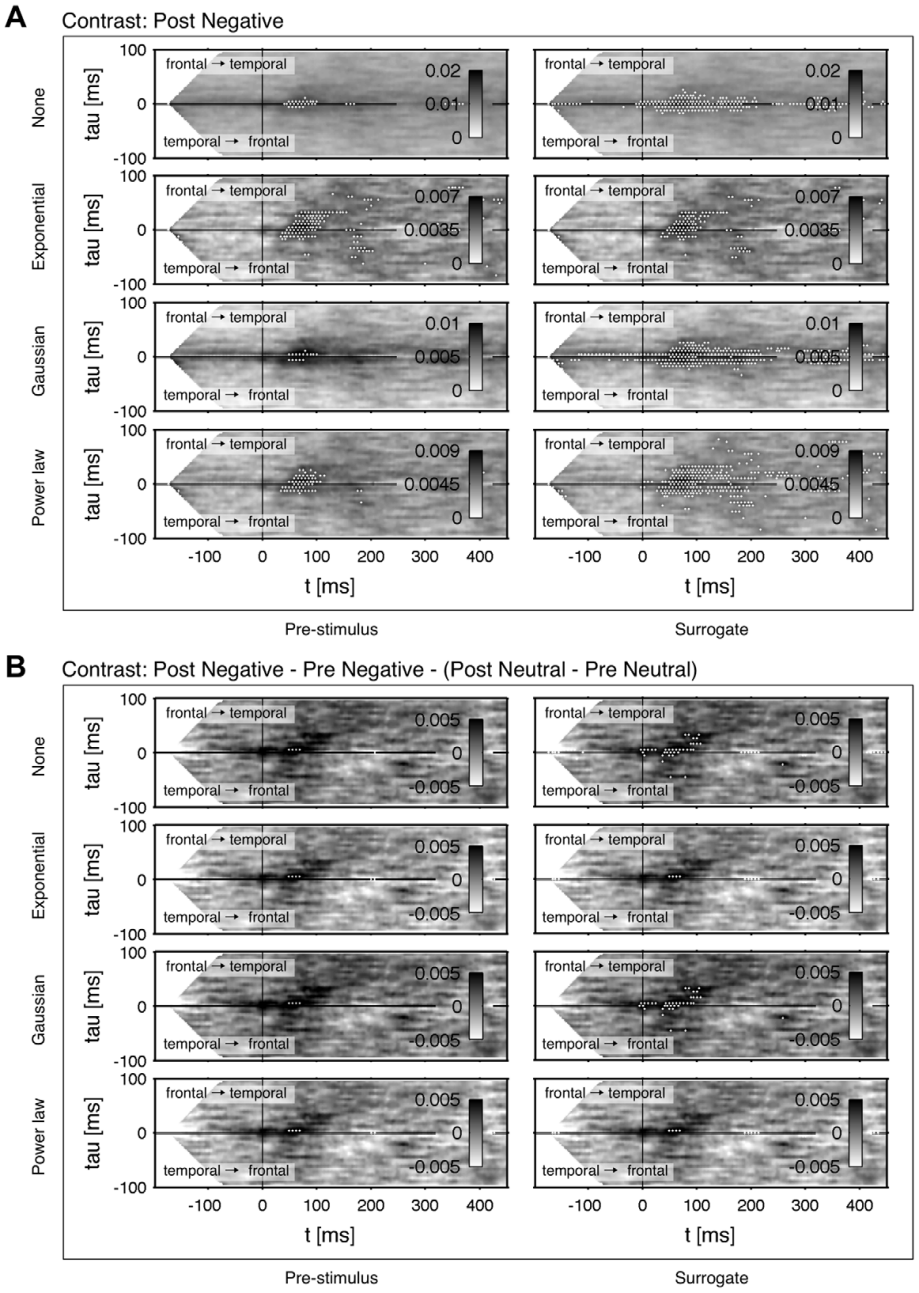
Impact of different types of de-correlations and baselines on a) the connectivity pattern *Post Negative* and b) the contrast *Post Negative - Pre Negative - (Post Neutral - Pre Neutral)*. Four types of de-correlation were implemented: *None* without any de-correlation, *Exponential* with 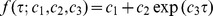, *Gaussian* with 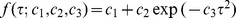 and *Power law* with 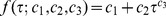. Data from the interval 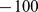
–0 ms provides a baseline for the FDR threshold in *Pre-Stimulus*. As an alternative approach *Surrogate* data was estimated by destroying correlation in phases. Significant higher connectivities are marked by white dots.

We also cross checked our results with surrogate data as a null distribution for the FDR. The surrogate data were generated by a randomization of the phase as suggested by [1]. A threshold based on the pre-stimulus seems more reliable and stable as the one estimated by surrogate data (Fig. 11A). As already discussed in the Methods (Mutual information of the phase) the pre-stimulus baseline is more conservative compared to surrogate data. Further, we tested several heuristic functions for the de-correlation step (exponential, Gaussian and power law) and found that the results in contrasts are almost identically Fig. 11B. When not using the contrast of two conditions an exponential or power law is preferable (see Fig. 11A). Compared to surrogate data the statistical outcome is more conservative, i.e. it is less sensitive to functional connections.

#### Conclusion of the MEG study

To conclude this section, we were able to measure the early significant connectivity between the frontal and temporal region as depicted in Fig. 10. Moreover, we successfully applied our proposed de-correlation technique of the Results (Pattern de-correlation) and additionally showed that the estimation of simple contrasts leads to satisfactory results with a meaningful interpretation: We could deal with mechanisms such as adaptation or fatigue. The meta-contrast, which includes important side-effects such as baseline activation and fatigue, contained a small but robust significant increase of synchronicity. Unfortunately, significant regions particular within the contrast patterns were small. Especially the contrast patterns suffered from a further reduced signal to noise ratio by subtracting signals. There are several reasons for the weak effect in functional connectivity: The experimental paradigm was designed in such a way, that the discrimination between the neutral and aversive stimuli was challenging for participants. The paradigm included the large number of 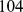 individual faces and lasted for about 60 min. Further, we had to deal with small data-sets, which consisted in average of 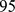 trials per subject and condition after pooling data from the lateral and frontal view. All in all, we were able to demonstrate that there is a significant increase in phase synchronization directed from the frontal to the temporal cortex at 59 ms. Descriptively, one can observe a tendency for a change in connectivity at about 130–190 ms, but this effect missed the threshold of significance.

## Discussion

### Summary

In our study we combined three crucial aspects into a universal and powerful tool for analyzing phase synchronization in terms of a functional connectivity among cortical activations in MEG. First, we implemented the mutual information of the phase as a measure of nonlinear phase synchronization [1] using efficient and adaptive techniques suggested by Kraskov et al. [2]. We further embedded the mutual information in our approach of connectivity patterns with a time and delay dimension [3]. Second, we proposed an efficient method to deal with correlated data which addresses an essential problem in MEG recordings. This de-correlation operates on the pattern level and was verified regarding its robustness on instantaneous noisy mixtures of synthetically generated data with an a-priori known connective structure. Third, we tested the compatibility of our analysis approach with the beamforming technique for MEG. This was done by the simulation of dipolar sources with a-priori known connectivity. In our simulations we also considered random brain noise and thermal sensor noise. We additionally suggested a noise suppression by a linear WMA filter as an intermediate step in the beamforming sequence. This combination increases the performance of the beamformer regarding the reconstruction of the underlying connectivity. Finally, our approach was applied on an MEG group study of Steinberg et al [32]. Although this data did not suite our requirements in an optimal way, we were able to uncover an increased synchronicity between frontal and temporal areas which confirmed the hypothesis of Steinberg and colleagues [32].

Because we are interested in the synchronicity with high temporal accuracy among neuronal populations in the human brain, non-invasive techniques such as MEG mark a good starting point. The phenomenon of synchronization plays a crucial role as a fundamental mechanism in neuronal communication [22]. In the exploration of synchronization we want to make as less assumptions as possible. On the one hand cognition can generally be understood as a nonstationary process, which is additionally impacted by intrinsic and instrumental noise. Therefore, the detection of synchronicity demands an average across trials. On the other hand the coupling mechanism as well as the spectral properties of the signals are assumed to be generally unknown, i.e. we take nonlinear interactions in the coupling into account and we consider all types of spectral properties from a peaked spectra to broad-banded spectral distributions. Both requirements – the estimation across trials as well as the analysis of a unknown dynamics – are respected by the mutual information of the phase. The mutual information is both sensitive to all types of interactions and it still converges with few data points if it is customized to ones needs. Therefore, we combined dedicated techniques as suggested by Kraskov et al. [2] with an additional moving time window. Besides the unknown types of interactions, we also did not assume any temporal properties, i.e. arbitrary underlying connective structures were addressed by a parametric time-delay following our previous suggestions [3].

The application of our approach on cortical activations from MEG recordings brought some further challenges: Inverse techniques are supposed to map the data back onto the cortex, but the reconstructed source waveforms contain correlations caused by an imperfect separation of the sensor data. Because we were interested in high reliability, we proposed a de-correlation method to remove partially mixed sources within the estimated connectivity patterns. Our de-correlation approach was efficient and easy to implement: For testing purposes the performance was evaluated with linear combinations of sources. We applied the baseline as a pre-stimulus interval assuming that it holds no underlying connectivities across the trials. Even a possible underlying connectivity within the pre-stimulus would be destroyed in a trial average, because the time interval between two stimulus on-sets is usually randomized. Therefore, the correlation within the pre-stimulus interval describes a decaying function regarding the time-delay, which is simply subtracted from the whole pattern to remove the influence of the instantaneous mixing. Heuristic exponential or power-law functions produced suitable results in the de-correlation procedure. We also compared our de-correlation to a source separation by ICA and found that our approach delivers higher stability and reliability in strongly correlated and noise-contaminated mixtures. Further, we claim that our de-correlation approach can be termed as an adaptive and universal method: It is adaptive because it is able do deal with arbitrary correlation lengths. The correlation length is typically determined by properties of the underlying process, e.g. specific frequencies or the intrinsic friction. The de-correlation is universal because it is independent of the underlying data, the method of phase synchronization and, when applied on patterns of reconstructed cortical sources, independent of the inverse technique.

### Phase lag index

We encountered the issue of volume conducting by the use of a dedicated de-correlation step of the proposed connectivity patterns. Techniques such as the phase lag index (PLI) and the improved version called weighted phase lag index (WPLI) also consider correlations caused by instantaneous mixtures [21], [75]. Both methods are basically modified versions of the phase coherence [19], [20]. The PLI as well as the WPLI measure the asymmetry of the phase difference among sources. The main idea is to make use of the fact that instantaneous mixtures exhibit a zero phase difference among non-delayed sources, i.e. a distribution of phase differences with a zero median is exclusively generated by a volume conduction and shall not contribute to the overall synchronization. In our method we explicitly compute time lagged interactions among sources without a restriction to zero delay values or a specific order of synchronization n∶m. We also do not explicitly exclude sources with a zero phase difference. Instead we gain knowledge of the impact of volume conducting by the help of a pre-stimulus baseline. However, the price of a higher generality is probably paid with higher computation time of the mutual information.

### Limitations

Next, we want to discuss some limitations of our proposed methods: As universal and versatile the mutual information is regarding the sensitivity to possible interactions on the one hand as non-specific it is on the other hand. If one is explicitly interested in the order of synchronization other techniques such as the ones proposed by [22], [23], [76] or [31] are beneficial, but lead to a higher dimension of the solution space. Because we followed a statistical approach, i.e. a synchronous regime at a specific time is detected by averaging over many repetitions, one implicitly assumes no changes of the underlying mechanisms across the trials. During data acquisition a decreasing level of vigilance might be a problem, if the processing strongly depends on such influences. Besides fatigue a subject might adapt to specific stimuli. In the Results (Application to MEG data) we already demonstrated that a contrast with a further control condition can help compensating effects of fatigue in a conditioning procedure. However, a contrast can equalize the level of synchronization but it cannot compensate a topological or temporal change of the involved network during the data acquisition.

So far, the statistical rating was implemented by a voxel-wise FDR on pattern level. A region-wise FDR control on a cluster level such as proposed by Chumbley and Friston [77], [78] could be applied on a pattern level as well as for a comparison across many patterns in an extended network to reduce the amount of false positive results.

Our proposed de-correlation step does not respect a background synchronicity caused by stationary resting state activities within the pre-stimulus interval. An increased synchronization caused by a resting state would result in a decreased and thus more conservative estimation of synchronization in the post-stimulus interval. Unfortunately, the pre-stimulus interval cannot be substituted effortlessly by a baseline using trial-shuffling as used e.g. by Vicente et al. and Wibral et al. [28], [79]. The reason is that the important auto-correlative part caused by the linear superposition in the instantaneous mixture is destroyed by the shuffling procedure. However, we found no pre-stimulus artifacts caused by a spurious resting state in the data of Steinberg and colleagues [32]. In future work it would be of relevance to investigate the quantitative impact of spurious resting state activities in the pre-stimulus interval on our proposed de-correlation step.

We followed a data-driven approach to avoid restrictive assumptions regarding the investigated process. As most of the data-driven approaches the mutual information belongs to the class of measures termed as functional connectivity. Such are conceptually limited to correlative statements, i.e. a common driver of two source results in a correlation among the two driven sources. In contrast, effective connectivity [47] enters a causal relationship as defined by Wiener [80]. For example two processes indexed with 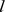 and 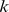 are said to be connected in a causal fashion, if including additional knowledge of the past of 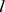 improves the prediction of the future of 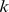. In this case information is transferred from 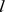 to 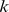. In the next section we will briefly discuss some methods measuring effective connectivity in MEG.

### Granger causality and dynamic causal modeling

In neuroscience the most popular model-based methods in quantifying effective connectivity can be categorized into two general classes, namely Granger causality (GC) and dynamic causal modeling (DCM). Such methods are capable of assessing a causal relationship across cortical sources. In the following we give a brief overview of the two methods and emphasize the differences to our approach. GC based techniques are defined either in the time-domain by a linear regression model [81] or in the frequency-domain by a spectral transfer function [82], [83]. Both approaches are generally restricted to linear interrelations and assume a stationarity of the underlying process. Further, GC is sensitive to a high level of noise [28], [84]. Sources including a memory structure cause problems in the case that they are partially correlated and differ in their channel noise. Especially, these constraints are typically present in imperfect source separations of EEG or MEG recordings and lead to false positive detections of causality [85]. As a parametric model-based approach the model has to be chosen carefully and has to be matched to the investigated system in terms of the underlying dynamics and its network topology. In case of model misspecification, a bias in causality might occur.

DCM was originally introduced by [69] in the context of causality analysis in fMRI. In contrast to GC, DCM is a more physiologically driven approach and belongs to the Bayesian framework. The main idea behind DCM is to choose the most plausible among a variety of generative models by the explanation of the observed data. To select the best among the competitive models the evidence is computed, which incorporates the accuracy and the complexity of the model [86]. The models are based on a network of distributed sources including physiologically motivated parameters [87]. Recently, DCM has also been expanded to MEG and EEG recordings [54], [88] by considering parametrized connections among and within sources and a neural-mass model based upon the Jansen-Rit model [52]. Such models are capable of generating event-related responses, but are much more sophisticated compared to models used for fMRI data. However, a DCM analysis demands prior information about the investigated system: First, the cortical dynamics has to be emulated explicitly in a physiologically realistic manner. So far, this is usually achieved by deterministic models. Because of the complexity of the system stochastic models including intrinsic brain noise should be taken into consideration [89]. Second, a family of competitive networks has to be predefined, i.e. the topology of sources and connections among them is hypothesis driven. Third, an input given by the stimulus has to be modeled as an explicit modulation of connections within the proposed system.

Both GC and DCM are capable of assessing the effective connectivity among cortical regions in terms of a causal relationship. But both method underlie strong model assumptions regarding the interrelations among sources and the topology. Especially the last aspect might be a problem in more exploratory studies. A data-driven approach, such as the one proposed in this work, could be used as a profitable complement to a model driven approach by supporting a pre-selection to match the model topology or as a prior in a Bayesian framework.

### Transfer entropy

With the mutual information we are able to detect nonlinear correlations shared among bivariate data which can be classified as a functional connectivity. In our approach the symmetry between driver and response is addressed by a parametric time-delay in terms of a delayed covariation among phases. Of course, with this approach a common driver leads to a false positive connection among two driven systems. As a measure of effective connectivity the transfer entropy (TE) is capable of detecting interactions in a causal sense as stated by Wiener [80]. It breaks the symmetry of a common driver by considering a conditional probability in the definition of the entropy, which includes past states of the system as an explicit side condition [90]. This expansion leads to a higher dimension of the probability density function resulting in a more challenging computation of its estimator. Gómez-Herrero et al. [91] generalized the approach of Kraskov et al. [2] for estimation of a time-dependent TE. They proved their approach with an autoregressive process (AR) and data from an electronic circuit based on two unidirectionally coupled nonlinear Mackey-Glass circuits. Vicente et al. tested the TE on instantaneous mixtures and applied it to MEG recordings on a sensor level of a self-paced finger lifting [28]. They demonstrated that the TE, which is formally a specific case of the mutual information, is capable of detecting strong nonlinear interactions such as quadratic or threshold nonlinearities in AR processes. Vicente et al. and Wibral et al. encounter the problem of partially correlated time-series, which reduce the specificity of the method, by comparing the TE of a shifted with a non-shifted time-series [28], [79]. Vakorin et al. address this issue with the help of a multivariate form. Their approach is based on a conditional mutual information which includes sources from the environment and leads to a more robust result than the bivariate approach [92]. In the studies of Wibral et al. [79] as well as Vakorin et al. [93] the TE was applied on reconstructed sources by using a broad-band time-domain beamformer. However, both studies did not focus on the signals' phases and instead applied a time-delayed embedding of the time-series in a higher dimensional space as proposed by Takens [94]. The combination of the TE of the phases could also be promising because this would allow the assessment of an directed synchronization and is thus in general robust against a common driving. Moreover, the application of the TE on the phase should be less demanding because on the one hand a smaller amount of data is required and on the other hand the TE on the phase could be implemented more easily. In general, the idea of a connectivity pattern could also allow an efficient de-correlation of the connectivity used in the framework of the TE.

### Conclusions and outlook

In this study we demonstrated that a customized measure of functional connectivity on the basis of the mutual information of the phase is applicable on MEG recordings. It allows the detection of phase synchronization without any assumptions regarding the underlying mechanisms. In particular we turned our attention on a time-dependency of the connectivity, which requires an efficient computation across trials. Following a data-driven way of analysis we decided to use FDR control in the pre-stimulus interval. Beside the statistical rating the pre-stimulus interval also allows an elegant and efficient way of removing correlations caused by an instantaneous mixing of the data. Because partial correlations are an inherent problem in MEG recordings our technique is of high practical relevance. We also verified our approach on simulations of MEG recordings to point out the feasibility of our method in combination with the beamforming technique. Finally, we tested our approach to an MEG group study in processing of emotionally relevant stimuli. Although the data was far from optimal regarding the requirements of our method, we were able to underpin a postulated connection between frontal and temporal region. To summarize, we contributed in closing the gap between methodical and practical aspects of the analysis of functional connectivity within the human brain. Our approach holds a lot of potential for future works: On the methodical side, our approach can be refined by expanding the estimator of mutual information with an estimator of transfer entropy, which allows the detection of an effective connectivity. On the experimental side, this form of analysis can be used for MEG recordings.
